# *O*-GlcNAc: Regulator of Signaling and Epigenetics Linked to X-linked Intellectual Disability

**DOI:** 10.3389/fgene.2020.605263

**Published:** 2020-11-23

**Authors:** Daniel Konzman, Lara K. Abramowitz, Agata Steenackers, Mana Mohan Mukherjee, Hyun-Jin Na, John A. Hanover

**Affiliations:** Laboratory of Cellular and Molecular Biology, National Institute of Diabetes and Digestive and Kidney Diseases, National Institutes of Health, Bethesda, MD, United States

**Keywords:** O-linked β-D-*N*-acetylglucosamine (*O*-GlcNAc), X-linked intellectual disability (XLID), epigenetics, histone modification, DNA methylation, nutrient-sensing

## Abstract

Cellular identity in multicellular organisms is maintained by characteristic transcriptional networks, nutrient consumption, energy production and metabolite utilization. Integrating these cell-specific programs are epigenetic modifiers, whose activity is often dependent on nutrients and their metabolites to function as substrates and co*-*factors. Emerging data has highlighted the role of the nutrient-sensing enzyme *O-*GlcNAc transferase (OGT) as an epigenetic modifier essential in coordinating cellular transcriptional programs and metabolic homeostasis. OGT utilizes the end-product of the hexosamine biosynthetic pathway to modify proteins with *O-*linked β-D-*N*-acetylglucosamine (*O-*GlcNAc). The levels of the modification are held in check by the *O-*GlcNAcase (OGA). Studies from model organisms and human disease underscore the conserved function these two enzymes of *O-*GlcNAc cycling play in transcriptional regulation, cellular plasticity and mitochondrial reprogramming. Here, we review these findings and present an integrated view of how *O-*GlcNAc cycling may contribute to cellular memory and transgenerational inheritance of responses to parental stress. We focus on a rare human genetic disorder where mutant forms of OGT are inherited or acquired *de novo*. Ongoing analysis of this disorder, OGT- X-linked intellectual disability (OGT-XLID), provides a window into how epigenetic factors linked to *O-*GlcNAc cycling may influence neurodevelopment.

## Introduction

Throughout the lifetime of an organism there is a requirement to be able to adapt to environmental changes, whether that be development, stress, or nutritional state. This adaptation requires changes in transcriptional programs that allow an appropriate gene regulatory network response. One way this is achieved is through epigenetic regulation: heritable modifications of DNA and histones that influence complex networks impacting transcription, DNA replication, and DNA repair (Janke et al., 2015). Importantly, the activity of all epigenetic modifying enzymes relies on the availability of specific metabolites. This sets up signaling pathways in which cells have evolved the ability to detect nutrient alterations and respond with epigenetic changes to coordinate appropriate transcriptional programs. Some of the most well defined metabolites and their influence on epigenetic modifications include: acetyl-CoA and histone acetylation; sirtuins, NAD^+^, and histone deacetylation; S-adenosylmethionine and DNA/histone methylation; FAD, α-ketoglutarate, and DNA/histone demethylation (Janke et al., 2015; Etchegaray and Mostoslavsky, 2016; Wong et al., 2017; Schvartzman et al., 2018). Here, we focus on the conserved epigenetic role that the nutrient-sensitive post-translational modification (PTM) *O-*GlcNAc, and the enzymes involved in *O-*GlcNAc cycling, the *O-*GlcNAc transferase (OGT) and the *O-*GlcNAcase (MGEA5 or OGA) have in coordinating transcriptional responses to environmental changes.

Neurodevelopment is one such process that is heavily coordinated by the crosstalk between the genome, environment and metabolic flux. Alterations during this process could lead to neurodevelopmental disorders, which constitute a broad range of disorders that originate during development of the central nervous system. As more and more sequences become available from neurodevelopmental disorder patients, it is becoming increasingly clear the essential role chromatin modifiers and epigenetic regulators play in these diseases. Of the hundreds of genes with mutations or copy number variations that are causative of neurodevelopmental disorders, chromatin regulation is the second most common association behind synaptic function (Gabriele et al., 2018). Interestingly, recent data has identified placental OGT levels as a biomarker for neurodevelopmental disorders (Howerton et al., 2013; Howerton and Bale, 2014) and mutations in the OGT gene as being causative to a subset of X-linked intellectual disability (XLID) patients (Pravata et al., 2020b).

**FIGURE 1:**
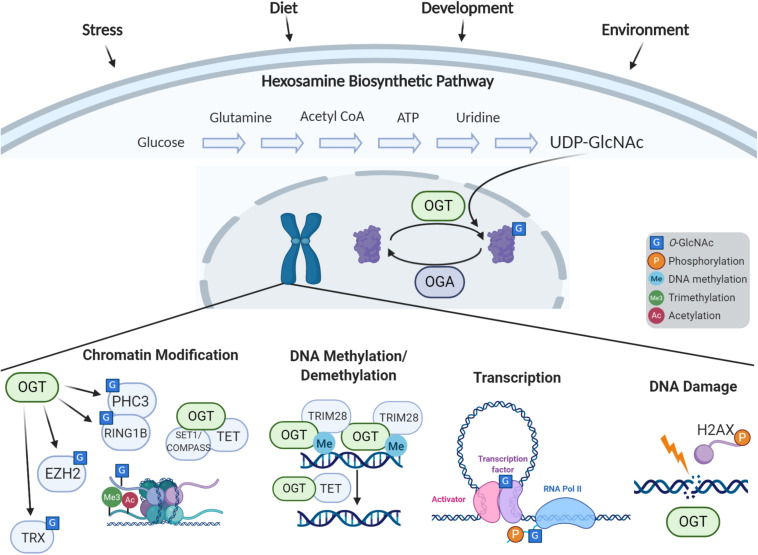
Cellular response to stress via *O-*GlcNAc-dependent epigenetic regulation. The hexosamine biosynthetic pathway incorporates nutrients and intermediate metabolites to form the nucleotide sugar UDP-GlcNAc. The *O*-GlcNAc Transferase (OGT) utilizes this nucleotide sugar as the sugar donor to modify diverse nucleocytoplasmic proteins including transcription factors, chromatin modifiers, and histones. This modification can be removed by *O*-GlcNAcase (OGA), whose levels are also implicated in regulation of gene expression. The process of *O*-GlcNAc cycling enables the cell to sense and integrate nutrient and signaling information and respond to extracellular stressors. *O*-GlcNAc influences histone modifications through the interactions of OGT with the Polycomb complex and SET1/COMPASS, OGA’s interactions with the trithorax complex, and direct *O*-GlcNAcylation of histones. OGT associates with TRIM28 to induce DNA methylation-dependent repression, and OGT is also involved in demethylation of DNA through the TET proteins. *O*-GlcNAc influences transcription by modification of transcription factors and the C-terminal domain of RNA Polymerase II. *O*-GlcNAc is also involved in the DNA damage response.

*O-*GlcNAc transferase uses the end-product of the hexosamine biosynthetic pathway (HBP), UDP-GlcNAc, to add a single GlcNAc monosaccharide onto serines and threonines of target intracellular proteins. To form UDP-GlcNAc, the HBP incorporates intermediate metabolites derived from carbohydrates, amino acids, fat, and nucleotides (Figure 1). Thus, OGT is uniquely positioned to sense environmental changes and respond through modifying key targets. A diverse array of more than 4,000 proteins have been identified to be *O*-GlcNAc modified (Ma and Hart, 2014), including transcription factors, epigenetic modulators, enzymes, kinases, mitochondrial proteins, structural proteins, nuclear porins, and components of vesicular trafficking pathways. How OGT recognizes specific target proteins remains relatively unclear, although it has preference for intrinsically disordered domains (Nishikawa et al., 2010). *O*-GlcNAcylation can influence modified proteins in various ways such as through crosstalk or competition with other PTMs like phosphorylation, altering enzyme activity, impacting protein stability, influencing subcellular localization, or altering binding partners (Bond and Hanover, 2013, 2015). Through this broad range of targets, *O-*GlcNAc influences many basic molecular processes including transcription, translation, proteostasis, and signaling [for more extensive reviews on the broad roles of *O*-GlcNAc and its targets see Bond and Hanover (2015) and Yang and Qian (2017)]. Regulation of *O-*GlcNAcylation has proven to be essential as too little or too much *O-*GlcNAc is associated with a number of diseases such as cancer, metabolic syndromes, and neurodegenerative diseases. Recently, OGT has been linked to a rare neurodevelopmental disorder known as OGT-XLID, which we hypothesize could be caused by dysfunction of the essential role of *O*-GlcNAc as an epigenetic regulator, the focus of this review.

OGT, OGA, and *O-*GlcNAc itself have all emerged as critical epigenetic regulators (Figure 1) essential for stem cell maintenance, development, and in the nervous system. Studies in model organisms have highlighted the conserved role OGT plays in gene regulation and cellular identity. Since the initial findings in *Drosophila* which defined the gene encoding OGT as a Polycomb group member critical for Hox gene silencing (Gambetta et al., 2009), *O-*GlcNAcylation has been recognized to play multifaceted roles in epigenetic regulation. Recent studies in *C. elegans* have demonstrated that OGT plays a critical role in preventing transitions between terminal cell fates. Advances in mass spectrometry technology have uncovered *O-*GlcNAcylation of histones, and is now widely believed to be part of the “histone code.” Studies have also indicated that *O-*GlcNAc plays a role in histone exchange and is essential for the DNA damage response (Na et al., 2020). Further, OGT has been found in complex with TET proteins, signifying a potential role in DNA demethylation (Chen et al., 2013; Deplus et al., 2013; Vella et al., 2013). *O-*GlcNac has also been defined to regulate transcription through modification of key transcription factors as well as the RNA polymerase II C-terminal repeat domain (Lewis and Hanover, 2014).

In this review we dissect the conserved and wide-ranging roles *O-*GlcNAc cycling has in regulating transcriptional networks which contribute toward maintaining cellular identity. We discuss how placental OGT could be a marker for placental stress contributing toward neurodevelopmental disorders. Lastly, we examine how OGT’s role in epigenetic regulation contributes toward mutations manifesting as an XLID in human patients.

## *O-*GlcNAc Cycling, Epigenetics and Cell Fate Determination: Lessons From Model Organisms

### Super Sex Combs: OGT and Hox Gene Repression

Across species, *O-*GlcNAc has a key role to play in development. Though its functions are numerous, OGT is particularly important in the spatiotemporal control of gene expression through its regulation of Hox genes. Conserved across bilaterians, the Hox genes encode critical transcription factors which specify the body plan along the anterior-posterior axis. Inappropriate regulation of these genes results in homeotic transformations, developmental errors in which one region of an animal inappropriately adopts the characteristics of another region. Two major groups of genes control the spatial expression of Hox genes: transcriptional repression by the Polycomb group (PcG) and activation by the trithorax group (trxG). In *Drosophila*, the gene encoding OGT was first described genetically by mutations that caused homeotic phenotypes including antenna-to*-*leg and wing-to*-*haltere transformations (Butler et al., 2019). Without knowing the enzymatic function of the protein it encodes, the gene was named *super sex combs (sxc)* for its loss of function phenotype. Like PcG genes *Polycomb (Pc)* and *extra sex combs (esc)*, *sxc* mutations affect the development of the sex combs, a structure on the forelimbs of *Drosophila* males. Similar to other components of the PcG, *sxc* was required for the repression of Hox genes in tissues where they should not be expressed. Derepression of Hox genes could explain the homeotic transformations associated with *sxc* mutations. Epistasis experiments confirmed this, as *sxc* flies lacking the Hox gene *Ubx* had more normal wing development (Ingham, 1984). Later, immunostaining of embryos showed aberrant expression of at least six PcG target genes including *Ubx* and *Abd-B* (Gambetta et al., 2009; Gambetta and Müller, 2014).

Experiments on the PcG gene *polyhomeotic (ph)* demonstrated genetic interactions with *sxc*. Flies carrying *sxc* and *ph* mutations had enhanced *sxc* phenotypes, with greater numbers of sex combs, stronger developmental defects, and lethality even earlier in development (Cheng et al., 1994). These findings uncovered further genetic interactions and demonstrated that *sxc* functions with the PcG to prevent ectopic expression of Hox genes.

In 2009, the protein product of *sxc* was determined to be the *O-*GlcNAc transferase, with expression of an *Ogt* transgene rescuing the pupal lethality of *sxc* mutant animals (Sinclair et al., 2009). Further studies better defined the interaction between *sxc/Ogt* and *ph*. In fact, co*-*IP experiments indicated that PH was *O-*GlcNAc modified (Gambetta et al., 2009). In extracts of larvae with homozygous *sxc/Ogt* mutations and no maternal *Ogt* contribution, PH was shown to aggregate into high molecular weight assemblies, which likely impaired its function (Gambetta and Müller, 2014). Loss of *O-*GlcNAcylation of PH resulted in *sxc* phenotypes, demonstrating modification was required to prevent aggregation, allowing PH to function with the PcG (Gambetta and Müller, 2014). Later, mass spectrometry of mouse embryonic stem cell (ESC) lysates demonstrated PHC3, the mammalian homolog of PH, is *O-*GlcNAc modified, suggesting the regulation of the PcG by *O*-GlcNAc is conserved (Myers et al., 2011). In human ESCs, the core subunit of PRC1, RING1B, is also modified and plays a role in targeting the complex (Maury et al., 2015). These findings clearly demonstrate the necessity of *O-*GlcNAc for the proper repression of Hox genes through the PcG component PH.

In mammals, the role of *O-*GlcNAc in PcG repression has diverged in certain ways. While PRC2 protein E(z) is not *O*-GlcNAc modified in *Drosophila* (Gambetta et al., 2009), several studies have identified its mammalian homolog EZH2 as modified, resulting in increased protein stability (Chu et al., 2014; Jiang et al., 2019). The effect of *O-*GlcNAc modification on PRC2-mediated H3K27me3 is controversial, with several publications describing a decrease associated with OGT depletion (Chu et al., 2014; Butler et al., 2019) and others noting no change (Myers et al., 2011; Forma et al., 2018; Jiang et al., 2019). These discrepancies likely arise from different cell lines being used, though one study found OGT knockdown prevented H3K27me3 changes associated with learning at specific gene promoters in mouse hippocampal tissue (Butler et al., 2019). This suggests the effects of *O-*GlcNAc may play roles in targeting PcG repression which are only apparent in certain developmental and genomic contexts.

*O-*GlcNAc transferase additionally interacts directly with the mammalian HOXA1 protein, as was found in a yeast-two*-*hybrid experiment (Lambert et al., 2012) and confirmed by co*-*IP (Draime et al., 2018). Though the authors did not find evidence for *O-*GlcNAc affecting localization, stability, or transcription factor activity (Draime et al., 2018), they only tested OGT and HOXA1 overexpression, so further experimentation may reveal additional layers of Hox gene regulation by *O-*GlcNAc. Although the phenotypes of *sxc/Ogt* flies are mostly consistent with those of other PcG genes, several findings suggest *sxc/Ogt* is also involved in Hox gene activation through trxG. Alone, both *sxc/Ogt* and *Asx* mutations produce anterior-to*-*posterior transformations associated with PcG mutations, but *sxc/Ogt*;*Asx* double heterozygotes display posterior-to*-*anterior transformations, which are typically associated with trxG mutations (Milne et al., 1999). This suggests *sxc/Ogt* acts not only as a Hox gene repressor through PcG but also as an activator through trxG. In fact, TRX and related transcriptional activators SET1/COMPASS and ASH1 have been shown to be *O-*GlcNAc modifiable, and staining for these proteins overlaps with *O-*GlcNAc on polytene chromosomes (Akan et al., 2016). The interaction between *O-*GlcNAc and trxG also appears in mammals, with *O-*GlcNAc modification of the H3K4 methyltransferase MLL5 working to stabilize this trxG enzyme (Ding et al., 2015). Thus, in addition to the necessary role of *O-*GlcNAc in the Polycomb repressive complex, this modification is also involved in transcriptional activation, highlighting the diverse routes in which *O-*GlcNAc regulates gene expression.

The appropriate spatiotemporal expression of Hox genes is crucial to the proper development of an organism. These regulatory systems which repress and activate Hox genes can cause severe developmental disorders when out of balance (Quinonez and Innis, 2014). Common clinical presentation for PcG mutations include intellectual disability and growth defects (Deevy and Bracken, 2019), which mirror those of OGT-XLID. Thus, the role of OGT in developmental regulation may contribute to symptoms of this disorder.

### O-GlcNAc Transferase in Cell Fate Plasticity

Developmental plasticity is necessary for the development of multicellular organisms, but this plasticity must be restricted when cells reach their terminal fates. Several labs have worked to define which factors are involved in the epigenetic processes that impose barriers between cell fates using the nematode *C. elegans*. The worm is a model system perfectly suited to study the genetics of development, as *C. elegans* are relatively simple multicellular animals, with a rigidly regulated series of cell divisions and fate decisions. Several studies have employed genetic screens to determine which genes are required to maintain proper cell fates. Two independent studies have found the sole nemotode *OGT* ortholog *ogt-1* is involved in the restriction of cellular plasticity.

Both experiments are based around genetic screens using strains sensitized to cell fate transformation by ectopic overexpression of the CHE-1 transcription factor. CHE-1 is a master regulator which is necessary to establish the fate of a specific pair of neurons called the ASE neurons, which can be monitored with a cell-specific reporter: *gcy-5::gfp* (Tursun et al., 2011). Due to mechanisms that impose barriers between cell fates, ectopic expression of *che-1* later in development does not have any effects on gene expression or cell fate. Even when overexpressed in tissues that would not normally transcribe *che-1*, neither activation of CHE-1 target gene expression nor induction of ASE fate are observed in otherwise wild-type animals (Tursun et al., 2011). However, they found that mutation of the histone chaperone *lin-53* allowed *che-1* overexpression to implement neural fate in germ cells (Tursun et al., 2011), suggesting a role for histones and potentially histone modifications in maintaining cellular identity. Using similar genetic systems, more recent studies have uncovered additional factors, including *ogt-1* (Hajduskova et al., 2019; Rahe and Hobert, 2019), that are required for maintenance of cellular identity.

Hajduskova et al. (2019) performed an RNAi screen of 730 candidate genes with known or predicted roles in chromatin modifications and remodeling. For this screen, expression of *che-1* was induced with a heat-shock promoter and reporter GFP signal was monitored. Ectopic GFP expression was detected with knockdown of 10 of the 730 tested genes. Different gene knockdowns allowed GFP expression in different tissues, suggesting tissue-specific regulatory mechanisms. This study focused on one hit in particular: *mrg-1*, an ortholog of the mammalian gene *MRG15*. Knockdown of *mrg-1* enabled *che-1* overexpression to reprogram germline cells to neuron-like cells. To further study the process by which MRG-1 works as a barrier between cell fates, the authors performed IP-MS to identify MRG-1 binding partners, and uncovered OGT-1. The IP-MS experiments also identified other known chromatin interactors such as *set-26* (H3K9 methyltransferase) and *sin-3* (histone deacetylase), which is itself a predicted interactor with OGT-1 (Yang et al., 2002).

A similar screen was performed in an independent lab which looked for genes involved in protecting epidermal cells from transdifferentiation (Rahe and Hobert, 2019). In this study, they overexpressed *che-1*, but restricted its expression to the epidermis starting at the end of the final larval stage (L4) and continuing into adulthood. Without mutagenesis, *gcy-5::gfp* expression was limited to ASE neurons, but mutations in seven genes caused ectopic expression of the reporter. Three independent missense mutations were isolated in the *ogt-1* gene. These three mutations were at highly conserved residues within the catalytic domains of OGT-1, in close proximity to sites previously reported to be necessary for enzymatic activity in human OGT (Lazarus et al., 2011). Other genes this screen identified included *dot-1.1* (an H3K79 methyltransferase) and *pmk-1*/p38-alpha MAPK. In mammalian neuroblastoma cells, OGT and p38 MAPK have been shown to physically interact to drive *O-*GlcNAcylation of specific targets (Cheung and Hart, 2008).

Though these studies were performed in nematodes in the context of ectopic gene expression, these results point toward an important role for *O-*GlcNAcylation in the control of cell fate plasticity. *ogt-1* came out of these two independent unbiased methods in the context of two different tissue types, suggesting it plays this role broadly. Though it has not been studied in as much depth in mammalian systems, some studies implicate *O-*GlcNAc in related processes. Much evidence points toward *O-*GlcNAc homeostasis being critical in stem and progenitor cells, which we will discuss further through its role in DNA damage and transcription factor regulation. In addition to its role restricting plasticity late in development, *O*-GlcNAc is critical in the networks of transcription factors which enable pluripotency in early development and adult stem cells which we will discuss in detail in its own section below. The developmental phenotypes of OGT-XLID patients and related transcriptomic data demonstrate the importance of *O*-GlcNAc in regulating sensitive cell fate specification events (Selvan et al., 2018). The importance of *O-*GlcNAc in cell fate may be intimately related to its roles in histone dynamics.

## *O-*GlcNAc Cycling and Histones

### Direct Modification of Histones by *O*-GlcNAc

*O*-GlcNAc affects chromatin and histone dynamics in a number of different ways. As described above, major complexes that modify histones are regulated by *O*-GlcNAc cycling. *O*-GlcNAc is also involved in the histone exchange required for DNA repair, and can modify histones directly.

Sakabe et al. (2010) first demonstrated direct evidence linking *O-*GlcNAcylation to the histone code, the holy grail of modern epigenetics (Jenuwein and Allis, 2001). Using an arduous combination of techniques they found *O-*GlcNAc on histones H2A, H2B, and H4. Acetylated histones were modified by *O-*GlcNAc and *O-*GlcNAcylation increased upon heat stress. Heat shock and OGT overexpression also modestly increased chromatin condensation. Using a chemical enrichment procedure, they mapped three *O-*GlcNAc sites on histones: H2AT101, H2BS36, and H4S47 (Sakabe et al., 2010). Additionally, they also provided evidence that other sites, including probable sites for modification of the remaining histone H3 must exist in the histone preparations. The identification of *O-*GlcNAc sites near known DNA interaction sites lead the authors to speculate that *O-*GlcNAcylation could induce major changes in chromatin structure not only by regulating peptide backbone conformation but also due to it being considerably larger than other common PTMs. One particularly intriguing *O-*GlcNAcylated site discussed was H4S47 (Sakabe et al., 2010). In yeast, mutation of this site to a cysteine induced activation of SWI/SNF targets independent of the SWI/SNF chromatin remodeling complex.

Since then, a total 17 different histone *O-*GlcNAcylation sites on H2A, H2AX, H2B, H3, H3.3, and H4 have been reported (see Table 1 for the full list with references). Evidence for these sites primarily comes from identification techniques such as chemoenzymatic detection, immunoblotting, selective enzymatic labeling, and lectin staining, in combinations with various mutation experiments. Due to the indirect nature of these methods, some skepticism has been raised about the existence of histone *O-*GlcNAcylation (Fujiki et al., 2011; Schouppe et al., 2011; Zhang et al., 2011; Hahne et al., 2012; Deplus et al., 2013; Lercher et al., 2015; Ronningen et al., 2015; Chen and Yu, 2016; Hirosawa et al., 2016; Hayakawa et al., 2017). However, the presence of *O-*GlcNAc at the two sites, H2AS40 and H3.3T32, have been confirmed based on the recognition of endogenous *O-*GlcNAc by tandem mass spectrometry (MS) analysis of histones isolated from mammalian cells (Sakabe et al., 2010; Fong et al., 2012), providing the most robust evidence for the *O*-GlcNAcylation of histones. Direct identification of peptidyl *O-*GlcNAcylated Ser/Thr by MS is burdensome due to its unstable nature, limiting attempts to confirm additional modification sites. Some studies have disputed the existence of histone *O*-GlcNAcylation, including one which reported an inability to detect modified histones in cultured mammalian cells (Gagnon et al., 2015; Gambetta and Muller, 2015). Still, indirect evidence suggests that *O-*GlcNAcylation is an important form of histone PTM.

**TABLE 1:** Reported sites of direct *O*-GlcNAc modification of histones.

Sites	Sample	Enrichment	Detection	Functions	References
H2AS40	mES cells	RP-HPLC and mAb	*O-*GlcNAc site by HCD tandem MS	Tightly relates with the differentiation in mouse trophoblast stem cells	Hirosawa et al., 2016; Hayakawa et al., 2017
H2AT101	HeLa cells	GalNAz labeling and DTT tagging	DTT tag by CID tandem MS	May be a part of the histone code	Sakabe et al., 2010
	Recombinant histone	*In vitro* reaction with OGT	*O-*GlcNAc site by ETD tandem MS	Facilitates H2BK120 monoubiquitination, for transcriptional activation	Fujiki et al., 2011
H2AXT101	HeLa cells	Laser micro-irradiation, immunofluorescence (IF) staining and microscope image acquisition	Abolished *O*-GlcNAc signal by CTD110.6 Ab for FLAG-tagged H2AXT139A mutant	The *O*-GlcNAcylation negatively regulates DNA double-strand break-induced phosphorylation of H2AX and MDC1 by restraining the expansion of these phosphorylation events from the sites of DNA damage.	Chen and Yu, 2016
H2AXS139				Co-localizes with DNA damage foci, may function in DNA damage repair	
H2BS36	HeLa cells	GalNAz labeling and DTT tagging	DTT tag by CID tandem MS	May be a part of the histone code	Sakabe et al., 2010
H2BS52	Various cell lines	Proteome-wide studies without any specific enrichment	Large scale CID tandem MS using the Oscore software, which assesses presence of *O*-GlcNAcylation	Suggests that *O*-GlcNAc and phosphorylation are not necessarily mutually exclusive but can occur simultaneously at adjacent sites.	Hahne et al., 2012
H2BS55					
H2BS56					
H2BS64	Calf thymus	Lectin-pulldown and butylamine tagging	Butylamine tagging by CID tandem MS	Suggest the presence of *O*-GlcNAc-modified proteins among the lectin-binding partners	Schouppe et al., 2011
H2BS112	Various cell lines	Immunofluorescence, Chromatin immunoprecipitation, Immunoblotting	Immunofluorescence, Chromatin immunoprecipitation, Immunoblotting	Preserves a stable chromatin and represses gene transcription at the early stage of adipocyte differentiation	Ronningen et al., 2015
	HEK293T and *Tet2* knockout mouse	HT-pulldown and Chromatin IP	IP	Direct physical link between OGT and TET2/3 proteins provide new insight into the regulation and function of OGT in the cell.	Deplus et al., 2013
	HeLa cell	*In vitro* reaction with OGT	ETD–MS/MS mapping	Facilitates H2BK120 monoubiquitination, for transcriptional activation	Fujiki et al., 2011
H3S91	Recombinant histone	*In vitro* reaction with OGT	*O-*GlcNAc site by ETD tandem MS	Preserves stable chromatin in the early stages of cell differentiation and may repress gene transcription in adipocytes	Fujiki et al., 2011
H3S112					
H3S123					
H3S10	HEK239 cell	Overexpression and IP by tag Ab	Abolished *O*-GlcNAc signal by lectin staining of FLAG-tagged H3S10A mutant	Competitively reduces the levels of H3S10 phosphorylation, therefore regulates the pathway that H3S10P involved in, such as passing the G2-M phase check point, regulating the H4K16ac	Zhang et al., 2011
H3.3T32	HeLa cell	IP by anti-H3 Ab	*O-*GlcNAc site by ETD tandem MS	Increases the phosphorylation of Thr32, Ser28, and Ser10, which are the specific mark of mitosis	Fong et al., 2012
H3.3T80	Calf thymus	Lectin-pulldown and butylamine tagging	Butylamine tag by CID tandem MS	Suggest the presence of *O*-GlcNAc-modified proteins among the lectin-binding partners	Schouppe et al., 2011
H4S47	HeLa cell	GalNAz labeling and DTT tagging	DTT tag by CID tandem MS	May be a part of the histone code	Sakabe et al., 2010

*mES cells, mouse Embryonic Stem cells; RP-HPLC, reversed-phase high performance liquid chromatography; Ab, antibody; mAb, monoclonal antibody; GalNAz, azide-modified galactose; DTT, dithiothreitol; IP, immunoprecipitation; MS, mass spectrometry analysis; HCD, higher-energy collisional dissociation; CID, collision-induced dissociation; ETD, electron-transfer dissociation.*

The 17 identified histone *O-*GlcNAcylation sites play various important roles in different biological functions. *O-*GlcNAc is identified at higher levels on H3 during interphase than mitosis, inversely related with phosphorylation, suggesting PTM crosstalk. Also, an increase in *O-*GlcNAcylation was observed to reduce mitosis specific phosphorylation at Ser10, Ser28, and Thr32. Inhibition of OGA hindered the transition from G2 to M phase of the cell cycle, showing a phenotype similar to hindering mitosis-specific phosphorylation on H3 delivering a mechanistic switch that orchestrates the G2-M transition of the cell cycle (Fong et al., 2012).

The combination of the extensive diversity of histone modifications allows for the complexity and flexibility of epigenetic regulation (Chen and Yu, 2016). It is now possible to map the genome- wide distribution and colocalization of histone modifications at high resolutions using ChIP-seq, revealing the many amalgamations of histone modification crosstalk, such as precondition, mutual exclusion, or coexistence (Pick et al., 2014). H2BS112 *O-*GlcNAcylation functions as a precondition for H2BK120 monoubiquitination, with GlcNAc acting as an anchor for ubiquitin ligase, ultimately resulting in transcriptional activation via H3K4me3 (Fujiki et al., 2011). An increase in the intracellular level of UDP-GlcNAc induces an increase in histone *O-*GlcNAcylation and a partial suppression in H3S10ph, suggesting these modifications are mutually exclusive. Further examples of competition between *O-*GlcNAcylation and phosphorylation have been reported for the H3T32, H3S10, and H2AXS139 sites (Zhang et al., 2011; Fong et al., 2012; Chen and Yu, 2016). Future studies should, therefore, investigate phosphorylation of other residues for which *O-*GlcNAc modification has been reported. The crosstalk between *O-*GlcNAcylation and acetylation should also be validated considering the presence of a HAT-like domain in OGA, although the enzymatic activity of this domain is a point of controversy (Torres and Hart, 1984; Toleman et al., 2006; Kim et al., 2007; Butkinaree et al., 2008; Hayakawa et al., 2013; Rao et al., 2013).

*O-*GlcNAcylation of site-specific adapter proteins directly regulate the stability of H2A/H2B dimers in the nucleosome in synthetic *O-*GlcNAcylated histones (Lercher et al., 2015). To generate homogenous *O*-GlcNAc modified nucleosomes, one study generated H2AT101 *O*-GlcNAc mimics by replacing the threonine with cysteine and using a series of chemical reactions *in vitro* to stably link GlcNAc to the thiol. The authors showed that H2AT101 GlcNAcylation destabilized the H3/H4 tetramer-H2A/B dimer interface reducing nucleosome stability. Thus, regulation of nucleosome stability by OGT-dependent GlcNAc transfer may contribute to transcriptional regulation. *O-*GlcNAc’s role in histone dynamics and in modifying variant histones make up an additional layer of histone regulation.

### H2AX and the DNA Damage Response

Histone modifications play a crucial role in chromatin organization through processes including DNA metabolism, replication, transcription, and repair. Modification and exchange of histones can also reorganize chromatin to allow DNA repair machinery to access damaged chromosomal DNA (Downs et al., 2004). H2AX is a histone variant that differs from H2A at various amino acid residues along the entire protein and in its C-terminal extensions (Bonisch and Hake, 2012). The importance of this histone variant is highlighted by the phenotypes of knockout mice, which show radiation sensitivity, developmental delay, and male infertility (Celeste et al., 2002). H2AX is a central player in the DNA damage response (DDR) when phosphorylated at serine 139 (γH2AX) (Wahl and Carr, 2001), and as mentioned previously, can also be *O-*GlcNAc modified at this site (Liu and Li, 2018). γH2AX is incorporated into nucleosomes at double strand break sites, where it promotes accumulation of DNA repair proteins (Wahl and Carr, 2001). γ-phosphorylation is an early event in the DSB damage response induced by the ATM and ATR kinases, which additionally activate kinases Chk1 and Chk2 (Wahl and Carr, 2001).

In the growth and development of an organism, DNA damage poses a serious risk, as mutations will propogate from progenitors to their daughter cells. To maintain genome integrity, the cell cycle is regulated by the DDR pathway following DNA damage stress, with *O-*GlcNAc involved by modifying the arrangement of histones and kinases (Hanover et al., 2018; Liu and Li, 2018). Blocking *O-*GlcNAc transferase activity leads to delayed DSB repair, reduced cell proliferation, and increased cell senescence *in vivo*, while increased *O-*GlcNAc promotes DSB repair and hyper-proliferation *in vivo* and *in vitro* (Efimova et al., 2019). These findings suggest *O-*GlcNAc is necessary to protect the genome and for proper cell cycle progression. These effects are likely related to OGT’s recruitment to sites of DNA damage, where it modifies H2A and mediator of DNA damage checkpoint 1 (MDC1) (Chen and Yu, 2016). In addition, one report has suggested OGT transfers GlcNAc onto H2AXS139, the same site as γ-phosphorylation (Chen and Yu, 2016). The authors suggest *O-*GlcNAc inhibits the DDR, though other studies suggest *O*-GlcNAc activates the DDR pathway (Efimova et al., 2019; Na et al., 2020).

**FIGURE 2:**
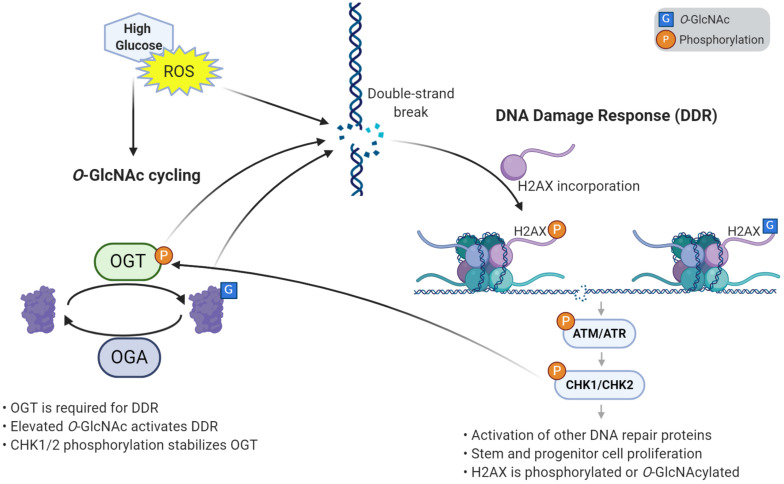
*O-*GlcNAcylation induces the DNA damage response and proliferation in stem cells. High glucose and reactive oxygen species (ROS) cause damage to DNA such as double strand breaks. *O-*GlcNAcylation is required to respond to DNA damage, through its accumulation at damage sites and activation of the DNA damage response (DDR) pathway. Response to DNA damage also requires the incorporation of variant histone H2AX, which is phosphorylated and can also be *O*-GlcNAc modified. Elevated *O-*GlcNAc induces stem and progenitor cell proliferation and the DNA damage response through γH2AX and a phosphorylation cascade of effector kinases. One of the downstream kinases, CHK1/2, phosphorylates OGT, which stabilizes the enzyme. This autoregulatory loop helps further promote the DNA damage response and proliferation.

The development of a multicellular organism is an energy-intensive and error-prone process. Stem and progenitor cells require high levels of glucose to grow and proliferate. A consequence of this energy use is the generation of reactive oxygen species (ROS), which cause damage, cellular stress, and DNA breaks. As such, DDR-related factors are prominent among proteins that accumulate *O-*GlcNAc when cells are stressed by ROS (Katai et al., 2016). Reciprocally, the DDR pathway has been shown to increase ROS levels (Rowe et al., 2008), pointing to a complex interplay between these processes. For example, high glucose has been demonstrated to elevate levels of *O-*GlcNAc, ROS, and DNA damage (Hu et al., 2019). The elevation of ROS in high glucose conditions may be linked to *O-*GlcNAc, as inhibition of OGT decreases ROS levels in a dose-dependent manner, and ultimately reduces neural tube defects in embryos of diabetic mice (Kim et al., 2017). Recently, we have reported that stress induces hyper-proliferation, *O-*GlcNAcylation, and DDR in *Drosophila* intestinal stem cells (Na et al., 2020). Likewise, genetic elevation of *O-*GlcNAc by deletion of *Oga* induced proliferation and DDR in fly intestinal stem cells, mouse embryonic fibroblasts, and mouse ESCs (Na et al., 2020). Previous work had shown that Chk1 phosphorylates OGT, which stabilizes the protein (Li et al., 2017). Through this interaction, we demonstrate that *O-*GlcNAc participates in an autoregulatory feedback loop where CHK1/CHK2 stabilizes OGT, allowing further *O-*GlcNAcylation that continues to activate the DDR pathway (Na et al., 2020) (Figure 2).

Thus, *O-*GlcNAcylation is a key regulator of the DDR pathway, which is crucial in supporting the development of a healthy organism. This further establishes *O-*GlcNAc’s role in cell identity discussed above, which will be explored below in our discussion of transcription factor modification. Observations of abnormal neural proliferation and developmental delay in mice harboring an *Oga* deletion in the brain (Olivier-Van Stichelen et al., 2017) suggest *O-*GlcNAc homeostasis is needed for the proper development of sensitive tissues such as the brain. Thus, the pathways discussed above may contribute to the developmental defects associated with OGT-XLID. Beyond the effects *O-*GlcNAc has on chromatin dynamics, *O-*GlcNAc interacts with DNA methylation pathways to regulate gene expression.

## OGT and DNA Methylation

DNA methylation is a critical epigenetic modification in mammals which occurs predominantly at the 5-position carbon on cytosine residues (5mC) followed by guanines. This epigenetic mark is involved in a variety of functions in the mammalian genome, including X-chromosome inactivation, gene silencing, genomic stability, cellular identity, and genomic imprinting (SanMiguel and Bartolomei, 2018). Two models for DNA methylation-dependent repression have been described. The first is a direct mechanism in which the presence of 5mC inhibits binding of transcription factors to DNA, thereby silencing gene expression. The second model is an indirect mechanism that involves recruitment of proteins that bind methylated DNA and associate with chromatin modifiers. These models are not mutually exclusive and can work in concert (Klose and Bird, 2006). Despite being stable and heritable, DNA methylation is also highly dynamic, particularly during development. Active DNA demethylation is mediated by the TET family proteins TET1, TET2, and TET3. These proteins iteratively oxidize 5mC to 5-hydroxymethylcytosine (5hmC), 5-formylcytosine (5fC), and 5-carboxylcytosine (5caC) (Kriaucionis and Heintz, 2009; Tahiliani et al., 2009; Ito et al., 2010, 2011; He et al., 2011). These modifications can be transient intermediates in the demethylation process which are ultimately removed by base excision repair (Cortellino et al., 2011; He et al., 2011), or can act on their own as a stable modifications (Bachman et al., 2014, 2015). Importantly, OGT is a well established binding partner of the TET proteins and has recently been described to play a critical role in DNA methylation-dependent repression.

Recent studies have revealed that OGT is found in complex with the three TET proteins. OGT was found to be associated with TET1 at gene promoters of transcriptionally active genes in mouse ESCs. This interaction was required for OGT binding to chromatin and enhanced TET1 activity (Vella et al., 2013). Further, *O-*GlcNAcylation of TET1 was shown to regulate TET1 stability (Shi et al., 2013a), and OGT enhanced TET1 activity *in vitro* (Hrit et al., 2018). Disrupting the OGT-TET1 interaction in mouse ESCs resulted in increased 5mC and compensatory increases in TET2, accompanied by transcriptional changes (Hrit et al., 2018). These data indicate that the TET1-OGT complex is critical for proper pluripotency gene regulatory networks which maintain stem cell identity.

TET2 and TET3 also form complexes with OGT. Interaction of TET2 with OGT associates at transcriptional start sites and facilitates histone *O-*GlcNAcylation. HCF-1 as part of the SET1/COMPASS complexes is a specific target of the TET2/3-OGT complex and promotes H3K4me3. A closer look at the TET3-OGT interaction indicated that this interaction stabilized OGT and enhanced chromatin association (Ito et al., 2014). However, another study found that *O-*GlcNAcylation of TET3 promoted its export from the nucleus, thereby inhibiting TET3 function (Zhang et al., 2014). Further complicating the regulation of TET proteins by OGT is the fact that they are also highly phosphorylated (Bauer et al., 2015) setting up the possibility of PTM crosstalk. While the functional relationship between OGT and TET proteins still remains controversial, there are some common themes in these papers. TET, OGT, and H2BS112 *O-*GlcNAc are colocalized in the genome, largely at CpG islands containing promoters of actively transcribed genes (Chen et al., 2013; Deplus et al., 2013; Vella et al., 2013). Thus this interaction likely reinforces active transcription to maintain cell intrinsic transcriptional program, possibly impacting development and gene expression in OGT-XLID.

In addition to its effects on the process of DNA demethylation, OGT has been found to be involved in gene silencing mediated by DNA methylation. OGT selectively associates with the scaffolding protein TRIM28 only in the presence of methylated DNA (Boulard et al., 2020). It has been proposed that *O*-GlcNAcylation of chromatin modifiers that interact with TRIM28 is required at the sites of retrotransposon promoters to repress their transcription (Boulard et al., 2020). This suggests disruption of *O*-GlcNAc cycling may lead to increased genome instability in addition to the contribution of impaired DNA damage response described above.

## Transcription Factor *O-*GlcNAcylation

**FIGURE 3:**
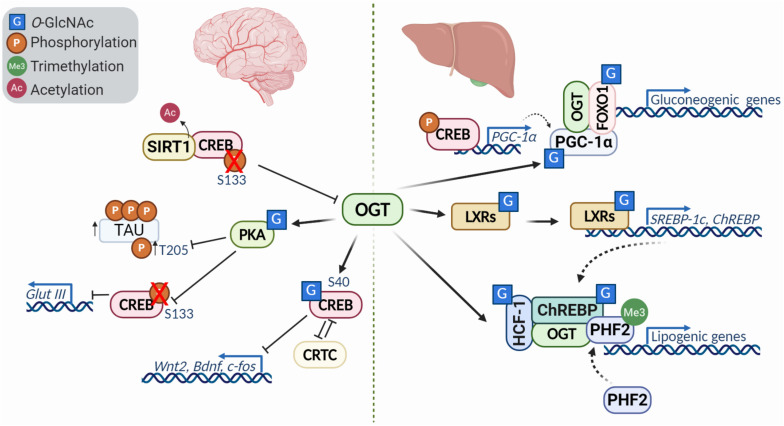
Diversity of transcription factor *O-*GlcNAcylation in the brain and liver. *O-*GlcNacylation of transcription factors can act to modulate their activity, localization, stability, binding partners and other post translational modifications. In the brain **(left)**, *O-*GlcNAcylation of CREB represses its activity and downregulates transcriptional targets like *Wnt2* and *Bdnf* in a CRTC (cAMP-regulated transcriptional co-activators)-dependent manner. Reduced *O-*GlcNAc of protein kinase A (PKA) is characteristic of Alzheimer’s disease, and leads to decreased phosphorylation of CREB at S133 and increased phosphorylation of tau at T205. Further, deacetylation of CREB by SIRT1, causes downregulation of OGT. In the liver **(right)**, modification of PGC-1a and association with OGT promotes FOXO1 *O-*GlcNAcylation, enhancing activity and promoting transcription gluconeogenic genes. The liver X receptors (LXRs) are nutrient sensors with critical roles in lipid metabolism, glucose homeostasis, and metabolism. *O-*GlcNAc modification of LXRs induces expression of SREBP-1 and ChREBP, key factors involved in hepatic lipogenesis and energy metabolism. HCF-1 *O-*GlcNAcylation stimulates OGT recruitment and modification of ChREBP, enhancing ChREBP activity and promoting recruitment of the activator PHF2 which binds the activating histone mark H3K4me3.

Gene expression is largely regulated by transcription factors, which themselves are heavily regulated by PTMs. *O-*GlcNAcylation is one of the major modifications that affects transcription factor functions, modulating their localization, stability, interacting partners, resulting in gene activation or silencing. While many studies have defined the role of *O-*GlcNAc modification of key transcription factors regulating processes like immune activation (Chang et al., 2020), here we focus on the role O-GlcNAc has in pluripotency and differentiation as well as in the nervous system and glucose and lipid metabolism in the liver as it relates more directly to neurodevelopmental disorders that will be described (Figure 3).

### *O*-GlcNAcylation in Development and Cell Differentiation

Throughout development, the process of differentiation allows a multicellular organism to form a complex system of multiple tissues and cell types. In adulthood, stem cells retain the capacity to differentiate allowing for tissue repair. *O-*GlcNAcylation during development is essential, as deletion of *Ogt* in mouse ESCs is lethal (Shafi et al., 2000). Further, reduced expression of *Ogt* disrupts stem cell self-renewal through deregulation of pluripotency transcriptional networks (Jang et al., 2012). This is likely due to OGT’s role in chromatin remodeling as described above, as well as through direct modification of transcription factors involved in pluripotency. In fact, the Yamanaka factors OCT4, SOX2, and C-MYC are all *O-*GlcNAc modified (Chou et al., 1995; Jang et al., 2012), and increasing *O-*GlcNAc in ESCs hampers normal differentiation (Jang et al., 2012). In fact, deleting *Oga* in the mouse is largely perinatal lethal (Keembiyehetty et al., 2015). Mice in which *Oga* was conditionally deleted in the brain exhibited microcephaly, high body fat percentage, hypotonia, and delayed development of the brain associated with abnormal cell proliferation and migration. This phenotype is related to transcriptional changes of pluripotency markers, including *Sox2*, *Nanog*, and *Otx2* (Olivier-Van Stichelen et al., 2017). The transcription factor *Sox2* and other transcription factors involved in the maintenance of mouse ESC pluripotency and stem cell self-renewal all require *O-*GlcNAcylation (Myers et al., 2011; Jang et al., 2012).

Cellular differentiation is regulated by *O-*GlcNAcylation, as lowered UDP-GlcNAc levels by HBP inhibition blocks the differentiation of adipocytes *in vitro* (Ishihara et al., 2010). Normal adipocyte differentiation in cell culture requires the transcription factors C/EBPα and C/EBPβ. It has been reported that C/EBPβ is modified by *O-*GlcNAc, and diminished *O-*GlcNAcylation reduces C/EBPα protein levels, suggesting the modification stabilizes the protein (Ishihara et al., 2010). Mass spectrometry analysis indicated two *O-*GlcNAc sites on Ser180 and Ser181, which are very close to C/EBPβ phosphorylation sites at Thr188, Ser184, and Thr179 (Li et al., 2009). The sequential phosphorylation of C/EBPβ at Thr188 then Ser184 by MAPK or CDK2, and Thr179 by GSK3β is required for DNA binding and transcriptional activity (Kim et al., 2007). Increased *O-*GlcNAcylation during adipocyte differentiation prevents C/EBPβ phosphorylation and subsequently delays adipocyte differentiation (Li et al., 2009). In addition, mutations of Ser180/181 rescued the phenotype induced by *O-*GlcNAcylation which suggests that the transcriptional activity of C/EBPβ is regulated by phosphorylation and *O-*GlcNAcylation in a competitive manner by alternative occupancy at adjacent sites (Li et al., 2009).

Hematopoietic stem cell (HSC) maintenance also requires balanced *O-*GlcNAc cycling. In fact, when *Oga* has been deleted in HSCs in mice, these mice exhibit diminished HSC pools as well as reduced intermediate progenitor populations. The elevated *O-*GlcNAcylation in progenitor cells of these mice were correlated with transcriptional changes in factors involved in adult stem cell maintenance, lineage specification and nutrient uptake (Abramowitz et al., 2019).

### Transcription Factor *O*-GlcNAcylation in the Nervous System

*O-*GlcNAcylation has been extensively studied in the brain due to its critical role during development described above, and in neurodegenerative diseases like Alzheimer’s (Griffith and Schmitz, 1995; Olivier-Van Stichelen et al., 2017). Emerging data has highlighted the role of transcription factor *O-*GlcNAcylation during neuronal development, synaptic plasticity and memory. CREB, a key transcription factor involved in learning and memory, is *O-*GlcNAc modified in the TAFII130 binding domain, a component of the TFIID transcriptional complex (Lamarre-Vincent and Hsieh-Wilson, 2003). *O-*GlcNAcylation of CREB impairs its interaction with TAFII130 and represses CREB transcriptional activity *in vitro* (Lamarre-Vincent and Hsieh-Wilson, 2003). Further studies demonstrated that *O-*GlcNAcylation of CREB at Ser40 modulates dendrite and axonal elongation with downregulation of Wnt2 and BDNF signaling (Rexach et al., 2012). It has also been suggested that glycosylation of CREB has a significant impact on long-term memory consolidation (Rexach et al., 2012). In accordance with this hypothesis, an independent research group has provided a link between *O-*GlcNAcylation, Protein Kinase A (PKA)-CREB signaling, and memory loss in Alzheimer’s disease. Alzheimer’s disease is associated with a decrease in *O-*GlcNAcylation, which has been shown to influence aggregating proteins such as tau (Akan et al., 2018). PKAs can be *O-*GlcNAc modified, which influences their localization, activity, and phosphorylation (Xie et al., 2016). The inhibition of PKA-CREB signaling by *O-*GlcNAcylation was associated with impaired learning and memory in mice.

While CREB itself is regulated by *O-*GlcNAc, it can also regulate OGT expression. CREB can be deactivated by the deacetylase SIRT1, thereby reducing OGT expression and promoting tau phosphorylation, one of the major events in the course of Alzheimer’s disease (Lu et al., 2020).

With important roles in regulating the transcriptional networks required for proper development, differentiation and within the nervous system, it is unsurprising that deregulation in *O-*GlcNAc homeostasis is associated with neurodevelopmental diseases.

### Transcription Factor *O*-GlcNAcylation in Carbohydrate and Lipid Metabolism

Beyond the brain, CREB also plays an important role in the liver where it regulates hepatic glucose and lipid metabolism (Herzig et al., 2001; Dentin et al., 2008). During prolonged fasting, CREB stimulates the gluconeogenic program with the coactivator PGC-1 (Herzig et al., 2001). PGC-1α (peroxisome proliferator-activated receptor gamma, co*-*activator 1 alpha) is a transcriptional co*-*activator that controls energy and nutrient homeostasis by coordinating gene expression. PGC-1α has been shown to form a complex with OGT and be *O-*GlcNAcylated at Ser333. Moreover, increased glucose levels lead to FOXO1 (Forkhead box other 1) *O-*GlcNAcylation via the PGC-1α/OGT complex, enhancing transcriptional activity (Housley et al., 2008). In addition, increased *O-*GlcNAc, either by addition of glucosamine or an OGA inhibitor, enhanced FOXO1 target gene expression in HepG2 cells (Kuo et al., 2008).

Insulin has two main functions within the liver: (1) downregulation of gluconeogenesis genes by initiating inhibitory phosphorylation of FOXO1, and (2) promotion of lipogenic pathways through activation of SHREBP-1c (Brown and Goldstein, 2008). *O-*GlcNAcylation regulates both pathways by attenuating insulin signaling and activating lipogenic pathways. The liver X receptors (LXRs) are described as nutritional sensors for lipid metabolism, glucose homeostasis and inflammation, and are posttranslationally modified by phosphorylation, acetylation, and *O-*GlcNAcylation (Anthonisen et al., 2010). Increased glucose levels leads to LXR *O-*GlcNAcylation, inducing SREBP-1c (sterol regulatory element binding protein 1c) expression. SREBP-1c is a major player of gene expression in hepatic lipogenesis (Anthonisen et al., 2010). LXR has been shown to interact and co*-*localize with OGT *in vitro* and *in vivo*. Additionally, LXR enhanced the expression of *SREBP-1c* and *ChREBP*α*/*β under hyperglycemic conditions (Bindesbøll et al., 2015). The effects of *O*-GlcNAc on the LXR pathway may extend beyond the liver, as this was the most dysregulated pathway found by transcriptomics of OGT-XLID mutant cells (Selvan et al., 2018). The LXR pathway has been shown to be critical in the development of dopaminergic neurons from stem cells (Ma et al., 2009), providing a possible link between this pathway and the developmental disorder.

ChREBP (carbohydrate responsive element binding protein) is a key factor of energy metabolism in the liver and is regulated by *O-*GlcNAcylation. High glucose or OGT expression increased ChREBP *O-*GlcNAcylation, stabilizing the protein and increasing expression of its target genes. *Oga* overexpression in mouse livers markedly reduced ChREBP *O-*GlcNAcylation and decreased abundance of ChREBP targets (Guinez et al., 2011). These results clearly demonstrated that *O-*GlcNAcylation of ChREBP increases its stability and activity. In addition, a recent study identified HCF-1 as a modulator of ChREBP activity and the lipogenic program, which is glucose dependent. Elevated glucose induced HCF-1 *O-*GlcNAcylation and HCF-1/ChREBP complex formation, where HCF-1 recruits OGT to further promote *O-*GlcNAcylation of ChREBP (Lane et al., 2019). Moreover, HCF-1/ChREBP complex formation was associated with the recruitment of epigenetic activator PHF2, which binds to H3K4me3 and enhances transcription (Lane et al., 2019). These data demonstrated that lipogenic gene expression is under the control of epigenetic modulations, ChREBP *O-*GlcNAcylation, and activation by HCF-1.

Through regulation of transcription factors, *O-*GlcNAc is able to alter patterns of gene expression in response to nutrient status and stress. Mutations in OGT may impair its targeting of specific pathways and disrupt their normal function in responding to the environment and coordinating development.

## Placental OGT as a Biomarker for Neurodevelopmental Disease: a Model of Transgenerational Inheritance

Prenatal development is a particularly vulnerable time, when tight regulation of transcriptional networks is required to transform a fertilized egg into a multicellular organism requiring complex tissue development. In mammals, energy flow from the mother to the fetus is mediated by the placenta, which transfers macronutrients, gases, and metabolites into fetal circulation. Thus, the placenta transmits nutritional and stress information from the mother to the developing fetus. Glucose is the primary fuel for the fetus, which is provided by maternal transfer through the placenta. The fetal brain is a particularly nutritionally demanding tissue during fetal development. Brain regions are more sensitive to nutrient availability at particular gestational stages. Interestingly, a recent hypothesis to explain the male-biased presentation of neurodevelopmental diseases focuses on sex differences in the placenta in relaying signals to the developing brain regarding maternal perturbations (Charil et al., 2010; Howerton and Bale, 2012, 2014; Gabory et al., 2013; Howerton et al., 2013; Davis and Pfaff, 2014; Nugent and Bale, 2015; Bale, 2016).

As an X-linked gene and having the ability to transmit nutritional information to the nucleus, OGT is a unique candidate to signal maternal stress through the placenta to the developing fetus in a sex-dependent manner. Interestingly, *O-*GlcNAcylation of placental proteins correlates with maternal glycemic index (Dela Justina et al., 2018). In fact, a genome-wide screen looking for sex-specific changes in placental transcription after exposure to early prenatal stress identified *Ogt* as a top candidate for exhibiting sexually dimorphic expression in the placenta and changes in expression as a response to maternal stress (Howerton et al., 2013). This study found that male mouse placentas had about half the amount of OGT protein, corresponding to decreased total *O-*GlcNAc levels and even lower levels of OGT and *O-*GlcNAc upon prenatal stress, as compared to their female counterparts (Howerton et al., 2013). Hemizygous and homozygous mice that had *Ogt* deleted specifically in the placenta recapitulated models of early prenatal stress presenting with hypothylamic mitochondrial dysfunction characterized by transcriptional changes and altered cytochrome c oxidase activity (Howerton and Bale, 2014).

As discussed previously, OGT plays a critical role in regulating gene expression, particularly as a key regulator of Polycomb repression. Analysis into how placental expression of *Ogt* could causally contribute to neurodevelopmental disorders in the offspring have highlighted OGT’s role in regulating the H3K27me3 repressive histone mark. Interestingly, Nugent et al. (2018), found that OGT establishes sex differences in placental H3K27me3, with an enrichment found in females. This sex difference was *Ogt* dependent, as genetic reduction of *Ogt* in mice masculinized female placental H3K27me3 levels (Nugent et al., 2018). It was hypothesized that the higher levels of H3K27me3 allow for resiliency and less transcriptional response to maternal stress in the female than the male placenta. One deregulated gene of particular interest was *Hsd17b3* (17-β-hydroxysteroid dehydrogenase-3). Through a ChIP-seq experiment on placental tissue, the researchers identified a correlation between *O*-GlcNAc occupancy and *Hsd17b3* expression and that *O*-GlcNAc is significantly reduced by exposure to early prenatal stress (Howerton and Bale, 2014). This gene codes a key enzyme in testosterone biosynthesis. Examination of testosterone in a model of early prenatal stress male placentas showed a significant reduction in testosterone levels, potentially contributing to hypothalamic changes in offspring (Howerton and Bale, 2014). Taken together, these observations supports the possibility that acting as an epigenetic modifier in the placenta, OGT is able to pass transgenerational stress signals from the mother to the offspring in a sex-specific manner.

## OGT and X-Linked Intellectual Disability (OGT-XLID): a Disease of *O-*GlcNAc Imbalance

**FIGURE 4:**
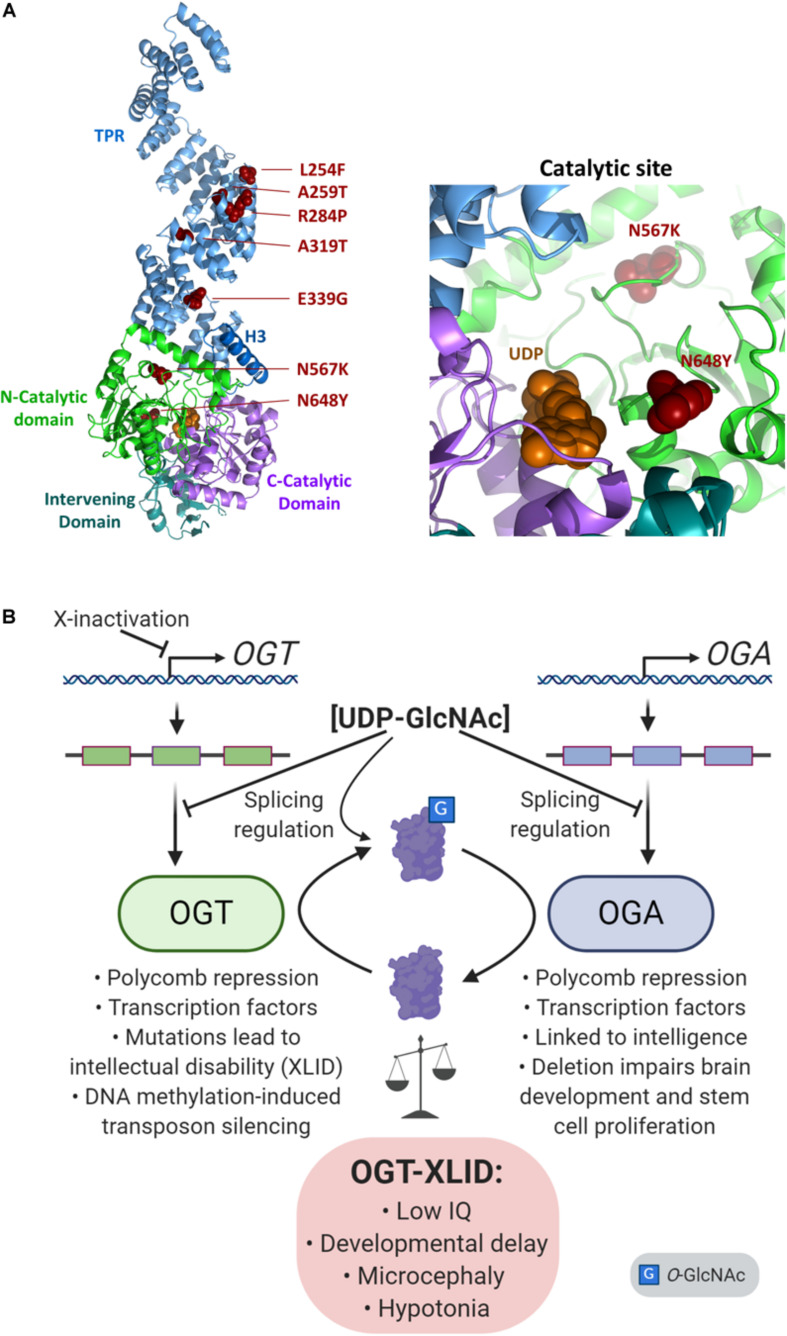
Imbalance of *O-*GlcNAc cycling leads to epigenetic changes and disease. **(A)** The presumed structure of OGT (Lazarus et al., 2011) shown as a cartoon in complex with UDP (orange spheres), with sites of OGT-XLID causative variants highlighted (red spheres). Each domain is colored-coded and labeled with its name. To better show the variants in the catalytic domain, a zoomed panel rotated 180° about the *y*-axis from the full structure is shown on the right. **(B)** Expression of both *OGT* and *OGA* are regulated by cellular concentrations of UDP-GlcNAc through splicing mechanisms. As an X-chromosome gene, *OGT* is also regulated by X-inactivation. OGT and OGA proteins dynamically cycle *O-*GlcNAc to regulate many epigenetic mechanisms such as the Polycomb repressive complexes and regulation of transcription factors. In addition, both genes are linked to intelligence: *OGT* mutations cause intellectual disability, and a GWAS meta-analysis found *OGA* is associated with intelligence (Savage et al., 2018). Deletion of *Oga* disrupts proper neural development and the proliferation of mouse embryonic stem cells (Olivier-Van Stichelen et al., 2017).

X-linked intellectual disabilities are a group of a neurodevelopmental disorders representing about 5-10% of all cases of intellectual disability (Pravata et al., 2020b). Over 200 genes have been linked to XLID, although some of the candidates remain controversial. Over the past few years, a syndromic form of XLID affecting multiple families has been described which co*-*segregates with variants in the human OGT gene (Vaidyanathan et al., 2017; Willems et al., 2017; Selvan et al., 2018; Pravata et al., 2019, 2020a, 2020b). At present, some 14 patients from 8 families have been analyzed which show non-synonymous variants in the OGT gene (Pravata et al., 2020a). All the patients carrying OGT variants were found to have decreased intellectual ability with IQ scores well below 70. In addition, the patients exhibit both mental and physical developmental delay, intrauterine growth retardation, low birth weight, short stature, restricted language skills, and drooling. A summary of XLID-causative variants and their position in the proposed OGT structure is shown in Figure 4A. It is notable that all of the inherited variants are in the TPR repeats (Bouazzi et al., 2015; Vaidyanathan et al., 2017; Willems et al., 2017; Selvan et al., 2018), which are implicated in both substrate binding and complex formation with other epigenetic regulators. Two mutations appeared *de novo* and are present in the catalytic domain, such as the N567L variant found in two female twins (Pravata et al., 2019), and the N648Y variant found in a male patient (Pravata et al., 2020a). On the basis of the common features of these patients and the fact that the clinical features co*-*segregate with the OGT gene it has been proposed that this syndrome be classified as a congenital disorder of glycosylation (CDG) termed OGT-CDG (Pravata et al., 2020b). Identification of this disorder strongly reinforces a growing body of evidence that *O-*GlcNAcylation plays a key role in development, particularly neurodevelopment. The involvement of OGT in numerous epigenetic pathways, with the propensity of epigenetic disorders to manifest in neurodevelopmental disorders (Gabriele et al., 2018) suggests possible mechanisms causing this form of XLID. Thus, the features of this disorder allows a dissection and discussion of the role of OGT and *O-*GlcNAcylation in many aspects of human physiology (Figure 4B).

### OGT Activity: Enzyme Stability, Target Recognition and Responsiveness to Hexosamine Flux

The previous examination of XLID patients have revealed changes in OGT activity and stability (Vaidyanathan et al., 2017; Willems et al., 2017; Selvan et al., 2018). Interestingly, several reports have suggested little to no change in *O-*GlcNAc levels (Vaidyanathan et al., 2017; Willems et al., 2017). One exception is the N648Y mutation which shows reduced enzymatic activity (Pravata et al., 2019). However, the tools we have available may be too insensitive to detect small changes or changes in subsets of substrates. In particular, the mutations associated with TPR repeats have the potential to affect substrate or complex recognition without direct effect on the catalytic domain. Our previous structural work on the TPRs allowed us to propose a role for an asparagine ladder motif in substrate recognition (Jinek et al., 2004). Recently this hypothesis was confirmed by additional structural studies (Levine et al., 2018). Thus substrate recognition may be affected by the XLID mutations.

The mutations in OGT may also destabilize the OGT protein. This was observed with the OGT L254F mutation in particular (Pravata et al., 2020a, b). However, current data reveal that while OGT-XLID variants may be destabilized, OGT protein levels in the majority of cell lines carrying the XLID variants were minimally altered (Pravata et al., 2020b). It is yet to be determined what effect these variants might have on levels of UDP-GlcNAc derived from hexosamine flux. Since OGT is a critical regulator of glucose utilization and metabolic reprogramming, changes in both glucose utilization and synthesis of UDP-GlcNAc may be altered in OGT-XLID (Saeed et al., 2016; Hanover et al., 2018).

### X-inactivation and XLID

The X chromosome has a unique pattern of inheritance compared to autosomal chromosomes. The X chromosome in males is inherited from the mother making recessive X-linked mutations predominant in males. The significance of the X-chromosomal location OGT has been previously discussed (Shafi et al., 2000; Hanover et al., 2003, 2012; Love et al., 2003, 2010; O’Donnell et al., 2004; Abramowitz et al., 2014; Olivier-Van Stichelen and Hanover, 2014, 2015; Olivier-Van Stichelen et al., 2014). In females, X-inactivation is primarily random, with individual cells inactivating one of the X chromosome employing a long non-coding RNA (*Xist*) and chromatin modifiers such as the Polycomb complexes (Love et al., 2010; Brockdorff, 2013; Froberg et al., 2013; Shi et al., 2013b; Monfort and Wutz, 2020). Interestingly, female XLID patients with *de novo* mutations at OGT N567K have been shown to exhibit extreme skewing in X-inactivation (98%) although it is unclear which of the two X chromosome are inactivated (Pravata et al., 2019). In these patients, the levels of *O-*GlcNAc are affected and this reduction can be recapitulated *in vitro* and in other model systems (Pravata et al., 2019). Given these findings, it is unclear why such skewing occurs in the context of this *de novo* mutation arising in female probands. OGT could contribute to DNA methylation/demethylation associated with the X-inactivation process. In addition, the role of OGT in Polycomb repression previously discussed raises the possibility that alterations in *O-*GlcNAc cycling could contribute to the skewing observed.

### The OGA Gene and Compensation for OGT Perturbations

In many instances, variants in OGT associated with XLID resulted in a reduction in levels of OGA (Vaidyanathan et al., 2017; Willems et al., 2017; Selvan et al., 2018; Pravata et al., 2020a, b). OGA can upregulate gene expression of *OGT* through activation of the transcription factor C/EBP-β (Vaidyanathan et al., 2017), in addition to this protein’s role in differentiation discussed above. Similarly, loss of *OGA* leads to an elevation in OGT protein levels (Keembiyehetty et al., 2015). The genes encoding OGA and OGT exhibit complex splicing patterns and recent findings suggest that mechanisms exist which serve to limit the translation of alternatively spliced species of the enzymes of *O-*GlcNAc cycling (Figure 4B) (Hanover et al., 2003; Park et al., 2017; Parra et al., 2020; Willems et al., 2017; Tan et al., 2020). *O-*GlcNAc regulates this process, suggesting a distinct feedback mechanism limiting the production of certain isoforms in response to *O-*GlcNAc elevation. In one study, an internal silencing site has been identified which appears to alter splicing of *OGT* mRNA, limiting its translation (Park et al., 2017). A similar mechanism is at play with OGA, where abundance of the enzyme is regulated by alternative splicing in response to *O*-GlcNAc levels (Tan et al., 2020). Thus, homeostatic systems are in place to limit the deregulation of total *O-*GlcNAc. However, lowering OGA levels can have a dramatic effect on neurodevelopment. We recently reported that mice in which *Oga* was deleted in the brain showed numerous phenotypes including microcephaly, enlarged ventricles, hypotonia, and developmental delay, strongly suggesting a possible link between OGT-XLID variants and perturbations of OGA levels (Olivier-Van Stichelen et al., 2017). In addition, a genome-wide association meta-analysis in 269,867 individuals identified *MGEA5/OGA* as one of the genes closely associated with intelligence (Savage et al., 2018). Studies on several OGT-XLID variants report a decrease in OGA levels (Vaidyanathan et al., 2017; Willems et al., 2017), although others have shown no evidence for a change in OGA levels (Selvan et al., 2018; Pravata et al., 2020a). So clear is this linkage between OGT variants and OGA that lowered OGA levels have been suggested as a diagnostic for XLID (Pravata et al., 2020b).

### XLID and Other OGT Functions: HCF-1 Cleavage

*O-*GlcNAc transferase strongly associates with HCF-1 and heavily modifies it. In combination, OGT-HCF-1 forms interactions with numerous epigenetic complexes (Lazarus et al., 2013; Janetzko et al., 2016; Kapuria et al., 2016, 2018; Levine and Walker, 2016; Leturcq et al., 2017; Vaidyanathan et al., 2017; Gao et al., 2018). In addition, OGT cleaves HCF-1 using a catalytic mechanism which has been recently studied (Lazarus et al., 2013; Bhuiyan et al., 2015; Janetzko et al., 2016; Kapuria et al., 2016, 2018). Some of the OGT variants show changes in HCF-1 cleavage, but this does not seem to be universal to the disorder (Vaidyanathan et al., 2017; Willems et al., 2017; Selvan et al., 2018; Pravata et al., 2020a, b). Mutations in the gene encoding HCF-1 have been found to cause another form of XLID (Koufaris et al., 2016), suggesting a possible overlap in the mechanism of these disorders. Unlike OGT-XLID, this disorder does not include obvious developmental and morphological abnormalities, but HCF-1 dysregulation may still be an important contributor to key features of OGT-XLID.

### OGT Interactions With Epigenetic Complexes

*O-*GlcNAc transferase interacts with numerous protein complexes associated with epigenetic regulation including the Polycomb repressive complexes, the pluripotency network associated with Oct4, Sin3A-HDAC complexes, and many others (Love et al., 2010; Hanover et al., 2012; Lewis and Hanover, 2014; Levine and Walker, 2016; Leturcq et al., 2017; Gao et al., 2018). The mutations seen in OGT-XLID could disrupt subsets of these interactions with accompanying changes in *O-*GlcNAc modification leading to a more pleiotropic deregulation of development. Improper regulation of these epigenetic complexes is known to impact development. For example, mutations in PRC2 proteins EZH2, SUZ12, and EED can cause Weaver Syndrome, an autism spectrum disorder that presents with developmental delay (Imagawa et al., 2017). These overlapping phenotypes may suggest common molecular pathways are at play with OGT-XLID.

At the present, it is difficult to examine these interactions quantitatively, but sensitive methods to examine both protein-protein interactions and *O-*GlcNAc turnover in those complexes are under continuous development.

## OGT and Neurodevelopmental Disease: Summary and Implications

The identification of OGT-XLID as a rare human disorder suggests that non-synonymous variants of OGT can be tolerated to a limited degree. These OGT variants are hypomorphic, leading to only modest changes in *O-*GlcNAc levels due to compensatory changes in the *O-*GlcNAcase expression. Patients with OGT-XLID show numerous developmental and neurodevelopmental deficits resulting in a form of intellectual disability. This intellectual disability phenotype results from changes in neurodevelopment which strongly suggests that *O-*GlcNAc addition and removal may play a particularly important role in the functioning and development of the brain. This is perhaps not surprising given the importance of glucose and its metabolites in the physiology of the central nervous system. In addition, the complexity of the human brain originates from a finely tuned developmental process influenced by both genome and environment (Gabriele et al., 2018). A particularly sensitive time of brain development occurs *in utero* when glucose is provided primarily by maternal transfer through the placenta. This underscores the importance of maternal nutritional and environmental state and proper placental glucose metabolism for fetal brain development.

Development of the human cortex is incredibly sensitive, in part due to the requirement for two waves of rapid proliferation of progenitor cells (Florio and Huttner, 2014). During these developmental periods, cells are particularly prone to the accumulation of genetic lesions. Errors in DNA replication and repair can induce single nucleotide variants and insertions-deletions (Ernst, 2016). Transposable elements are another major source of genome instability which have been linked to neurological disorders (Doyle et al., 2017). Mutations in OGT may derepress transposable elements considering the recent findings of OGT working with TRIM28 to silence retrotransposons in a DNA methylation-dependant manner (Boulard et al., 2020). TRIM28 silencing is active in neural progenitor cells, and heterozygous mice present behavioral phenotypes which suggest an important role in brain development (Whitelaw et al., 2010; Grassi et al., 2019). The roles OGT plays both in activating the DNA damage response and in the silencing of retrotransposons help maintain genome integrity in the developing brain. The contribution of DNA damage and transposable element activity to OGT-XLID phenotypes should be investigated further.

Thus, the functional and morphological features of the human brain render it highly vulnerable to both genetically and environmentally induced alterations. In addition, there is an increasing awareness that chromatin regulation may be central to understanding neurodevelopmental disorders (Gabriele et al., 2018). The insights gained from analysis of both OGT-XLID patients and deregulated placental OGT, are likely to provide a platform for understanding how the *O-*GlcNAc pathway is integrated in human physiology. We have highlighted the role of *O*-GlcNAc cycling in numerous epigenetic complexes regulating development and differentiation. We have also examined the role of *O*-GlcNAc cycling in stem cell differentiation and the regulation of DNA damage response signaling. Finally, we have argued that compensatory mechanisms may be in place to limit the impact of the OGT mutations including X-inactivation of OGT, intron retention of OGT transcripts, OGA down regulation by Polycomb repression, intron retention, and transcription. These highly varied modes of regulation serve to buffer the effects of the OGT mutations, but may themselves have phenotypic consequences.

## Author Contributions

DK, LA, and JH edited the manuscript. All authors contributed to the literature search, wrote specific sections of the manuscript, and agreed on the final version.

## Conflict of Interest

The authors declare that the research was conducted in the absence of any commercial or financial relationships that could be construed as a potential conflict of interest.

## References

[B1] Abramowitz L. Olivier-Van Stichelen S. Hanover J. (2014). Chromosome imbalance as a driver of sex disparity in disease. *J. Genomics* 2 77–88. 10.7150/jgen.8123 25031659 PMC4091450

[B2] Abramowitz L. K. Harly C. Das A. Bhandoola A. Hanover J. A. (2019). Blocked O-GlcNAc cycling disrupts mouse hematopoeitic stem cell maintenance and early T cell development. *Sci. Rep.* 9:12569. 10.1038/s41598-019-48991-8 31467334 PMC6715813

[B3] Akan I. Love D. C. Harwood K. R. Bond M. R. Hanover J. A. (2016). Drosophila O-GlcNAcase Deletion Globally Perturbs Chromatin O-GlcNAcylation. *J. Biol. Chem.* 291 9906–9919. 10.1074/jbc.M115.704783 26957542 PMC4858994

[B4] Akan I. Olivier-Van Stichelen S. Bond M. R. Hanover J. A. (2018). Nutrient-driven O-GlcNAc in proteostasis and neurodegeneration. *J. Neurochem.* 144 7–34. 10.1111/jnc.14242 29049853 PMC5735008

[B5] Anthonisen E. H. Berven L. Holm S. Nygard M. Nebb H. I. Gronning-Wang L. M. (2010). Nuclear receptor liver X receptor is O-GlcNAc-modified in response to glucose. *J. Biol. Chem.* 285 1607–1615. 10.1074/jbc.M109.082685 19933273 PMC2804318

[B6] Bachman M. Uribe-Lewis S. Yang X. Burgess H. E. Iurlaro M. Reik W. (2015). 5-Formylcytosine can be a stable DNA modification in mammals. *Nat. Chem. Biol.* 11 555–557. 10.1038/nchembio.1848 26098680 PMC5486442

[B7] Bachman M. Uribe-Lewis S. Yang X. Williams M. Murrell A. Balasubramanian S. (2014). 5-Hydroxymethylcytosine is a predominantly stable DNA modification. *Nat. Chem.* 6 1049–1055. 10.1038/nchem.2064 25411882 PMC4382525

[B8] Bale T. L. (2016). The placenta and neurodevelopment: sex differences in prenatal vulnerability. *Dialogues Clin. Neurosci.* 18 459–464. 10.31887/dcns.2016.18.4/tbale 28179817 PMC5286731

[B9] Bauer C. Göbel K. Nagaraj N. Colantuoni C. Wang M. Müller U. (2015). Phosphorylation of TET proteins is regulated via O-GlcNAcylation by the O-linked N-acetylglucosamine transferase (OGT). *J. Biol. Chem.* 290 4801–4812. 10.1074/jbc.M114.605881 25568311 PMC4335217

[B10] Bhuiyan T. Waridel P. Kapuria V. Zoete V. Herr W. (2015). Distinct OGT-Binding Sites Promote HCF-1 Cleavage. *PLoS One* 10:e0136636. 10.1371/journal.pone.0136636 26305326 PMC4549301

[B11] Bindesbøll C. Fan Q. Nørgaard R. C. MacPherson L. Ruan H. B. Wu J. (2015). Liver X receptor regulates hepatic nuclear O-GlcNAc signaling and carbohydrate responsive element-binding protein activity. *J. Lipid Res.* 56 771–785. 10.1194/jlr.M049130 25724563 PMC4373736

[B12] Bond M. R. Hanover J. A. (2013). O-GlcNAc cycling: a link between metabolism and chronic disease. *Annu. Rev. Nutr.* 33 205–229. 10.1146/annurev-nutr-071812-161240 23642195 PMC10483992

[B13] Bond M. R. Hanover J. A. (2015). A little sugar goes a long way: the cell biology of O-GlcNAc. *J. Cell Biol.* 208 869–880. 10.1083/jcb.201501101 25825515 PMC4384737

[B14] Bonisch C. Hake S. B. (2012). Histone H2A variants in nucleosomes and chromatin: more or less stable? *Nucleic Acids Res.* 40 10719–10741. 10.1093/nar/gks865 23002134 PMC3510494

[B15] Bouazzi H. Lesca G. Trujillo C. Alwasiyah M. K. Munnich A. (2015). Nonsyndromic X-linked intellectual deficiency in three brothers with a novel MED12 missense mutation [c.5922G>T (p.Glu1974His)]. *Clin. Case Rep.* 3 604–609. 10.1002/ccr3.301 26273451 PMC4527805

[B16] Boulard M. Rucli S. Edwards J. R. Bestor T. H. (2020). Methylation-directed glycosylation of chromatin factors represses retrotransposon promoters. *Proc. Natl. Acad. Sci. U.S.A.* 117 14292–14298. 10.1073/pnas.1912074117 32522876 PMC7322000

[B17] Brockdorff N. (2013). Noncoding RNA and Polycomb recruitment. *RNA* 19 429–442. 10.1261/rna.037598.112 23431328 PMC3677253

[B18] Brown M. S. Goldstein J. L. (2008). Selective versus total insulin resistance: a pathogenic paradox. *Cell Metab.* 7 95–96. 10.1016/j.cmet.2007.12.009 18249166

[B19] Butkinaree C. Cheung W. D. Park S. Park K. Barber M. Hart G. W. (2008). Characterization of beta-N-acetylglucosaminidase cleavage by caspase-3 during apoptosis. *J. Biol. Chem.* 283 23557–23566. 10.1074/jbc.M804116200 18586680 PMC2527095

[B20] Butler A. A. Sanchez R. G. Jarome T. J. Webb W. M. Lubin F. D. (2019). O-GlcNAc and EZH2-mediated epigenetic regulation of gene expression during consolidation of fear memories. *Learn. Mem.* 26 373–379. 10.1101/lm.049023.118 31416910 PMC6699408

[B21] Celeste A. Petersen S. Romanienko P. J. Fernandez-Capetillo O. Chen H. T. Sedelnikova O. A. (2002). Genomic instability in mice lacking histone H2AX. *Science* 296 922–927. 10.1126/science.1069398 11934988 PMC4721576

[B22] Chang Y. H. Weng C. L. Lin K. I. (2020). O-GlcNAcylation and its role in the immune system. *J. Biomed. Sci.* 27:57 10.1186/s12929-020-00648-9 PMC7189445 32349769

[B23] Charil A. Laplante D. P. Vaillancourt C. King S. (2010). Prenatal stress and brain development. *Brain Res. Rev.* 65 56–79. 10.1016/j.brainresrev.2010.06.002 20550950

[B24] Chen Q. Chen Y. Bian C. Fujiki R. Yu X. (2013). TET2 promotes histone O-GlcNAcylation during gene transcription. *Nature* 493 561–564. 10.1038/nature11742 23222540 PMC3684361

[B25] Chen Q. Yu X. (2016). OGT restrains the expansion of DNA damage signaling. *Nucleic Acids Res.* 44 9266–9278. 10.1093/nar/gkw663 27458206 PMC5100584

[B26] Cheng N. N. Sinclair D. A. Campbell R. B. Brock H. W. (1994). Interactions of polyhomeotic with Polycomb group genes of Drosophila melanogaster. *Genetics* 138 1151–1162. 7896097 10.1093/genetics/138.4.1151 PMC1206254

[B27] Cheung W. D. Hart G. W. (2008). AMP-activated protein kinase and p38 MAPK activate O-GlcNAcylation of neuronal proteins during glucose deprivation. *J. Biol. Chem.* 283 13009–13020. 10.1074/jbc.M801222200 18353774 PMC2435304

[B28] Chou T. Y. Dang C. V. Hart G. W. (1995). Glycosylation of the c-Myc transactivation domain. *Proc. Natl. Acad. Sci. U.S.A.* 92 4417–4421. 10.1073/pnas.92.10.4417 7753821 PMC41955

[B29] Chu C. S. Lo P. W. Yeh Y. H. Hsu P. H. Peng S. H. Teng Y. C. (2014). O-GlcNAcylation regulates EZH2 protein stability and function. *Proc. Natl. Acad. Sci. U.S.A.* 111 1355–1360. 10.1073/pnas.1323226111 24474760 PMC3910655

[B30] Cortellino S. Xu J. Sannai M. Moore R. Caretti E. Cigliano A. (2011). Thymine DNA glycosylase is essential for active DNA demethylation by linked deamination-base excision repair. *Cell* 146 67–79. 10.1016/j.cell.2011.06.020 21722948 PMC3230223

[B31] Davis E. P. Pfaff D. (2014). Sexually dimorphic responses to early adversity: implications for affective problems and autism spectrum disorder. *Psychoneuroendocrinology* 49 11–25. 10.1016/j.psyneuen.2014.06.014 25038479 PMC4165713

[B32] Deevy O. Bracken A. P. (2019). PRC2 functions in development and congenital disorders. *Development* 146:dev181354. 10.1242/dev.181354 31575610 PMC6803372

[B33] Dela Justina V. Dos Passos Junior R. R. Bressan A. F. Tostes R. C. Carneiro F. S. Soares T. S. (2018). O-linked N-acetyl-glucosamine deposition in placental proteins varies according to maternal glycemic levels. *Life Sci.* 205 18–25. 10.1016/j.lfs.2018.05.013 29746846

[B34] Dentin R. Hedrick S. Xie J. Yates J. III Montminy M. (2008). Hepatic glucose sensing via the CREB coactivator CRTC2. *Science* 319 1402–1405. 10.1126/science.1151363 18323454

[B35] Deplus R. Delatte B. Schwinn M. K. Defrance M. Mendez J. Murphy N. (2013). TET2 and TET3 regulate GlcNAcylation and H3K4 methylation through OGT and SET1/COMPASS. *EMBO J.* 32 645–655. 10.1038/emboj.2012.357 23353889 PMC3590984

[B36] Ding X. Jiang W. Zhou P. Liu L. Wan X. Yuan X. (2015). Mixed Lineage Leukemia 5 (MLL5) Protein Stability Is Cooperatively Regulated by O-GlcNac Transferase (OGT) and Ubiquitin Specific Protease 7 (USP7). *PLoS One* 10:e0145023. 10.1371/journal.pone.0145023 26678539 PMC4683056

[B37] Downs J. A. Allard S. Jobin-Robitaille O. Javaheri A. Auger A. Bouchard N. (2004). Binding of chromatin-modifying activities to phosphorylated histone H2A at DNA damage sites. *Mol. Cell* 16 979–990. 10.1016/j.molcel.2004.12.003 15610740

[B38] Doyle G. A. Crist R. C. Karatas E. T. Hammond M. J. Ewing A. D. Ferraro T. N. (2017). Analysis of LINE-1 Elements in DNA from Postmortem Brains of Individuals with Schizophrenia. *Neuropsychopharmacology* 42 2602–2611. 10.1038/npp.2017.115 28585566 PMC5686486

[B39] Draime A. Bridoux L. Belpaire M. Pringels T. Degand H. Morsomme P. (2018). The O-GlcNAc transferase OGT interacts with and post-translationally modifies the transcription factor HOXA1. *FEBS Lett.* 592 1185–1201. 10.1002/1873-3468.13015 29465778

[B40] Efimova E. V. Appelbe O. K. Ricco N. Lee S. S. Liu Y. Wolfgeher D. J. (2019). O-GlcNAcylation enhances double-strand break repair, promotes cancer cell proliferation, and prevents therapy-induced senescence in irradiated tumors. *Mol. Cancer Res.* 17 1338–1350. 10.1158/1541-7786.mcr-18-1025 30885991 PMC6548675

[B41] Ernst C. (2016). Proliferation and differentiation deficits are a major convergence point for neurodevelopmental disorders. *Trends Neurosci.* 39 290–299. 10.1016/j.tins.2016.03.001 27032601

[B42] Etchegaray J. P. Mostoslavsky R. (2016). Interplay between metabolism and epigenetics: a nuclear adaptation to environmental changes. *Mol. Cell* 62 695–711. 10.1016/j.molcel.2016.05.029 27259202 PMC4893201

[B43] Florio M. Huttner W. B. (2014). Neural progenitors, neurogenesis and the evolution of the neocortex. *Development* 141 2182–2194. 10.1242/dev.090571 24866113

[B44] Fong J. J. Nguyen B. L. Bridger R. Medrano E. E. Wells L. Pan S. (2012). beta-N-Acetylglucosamine (O-GlcNAc) is a novel regulator of mitosis-specific phosphorylations on histone H3. *J. Biol. Chem.* 287 12195–12203. 10.1074/jbc.M111.315804 22371497 PMC3320971

[B45] Forma E. Jóźwiak P. Ciesielski P. Zaczek A. Starska K. Bryś M. (2018). Impact of OGT deregulation on EZH2 target genes FOXA1 and FOXC1 expression in breast cancer cells. *PLoS One* 13:e0198351. 10.1371/journal.pone.0198351 29864144 PMC5986130

[B46] Froberg J. Yang L. Lee J. (2013). Guided by RNAs: X-inactivation as a model for lncRNA function. *J. Mol. Biol.* 425 3698–3706. 10.1016/j.jmb.2013.06.031 23816838 PMC3771680

[B47] Fujiki R. Hashiba W. Sekine H. Yokoyama A. Chikanishi T. Ito S. (2011). GlcNAcylation of histone H2B facilitates its monoubiquitination. *Nature* 480 557–560. 10.1038/nature10656 22121020 PMC7289526

[B48] Gabory A. Roseboom T. J. Moore T. Moore L. G. Junien C. (2013). Placental contribution to the origins of sexual dimorphism in health and diseases: sex chromosomes and epigenetics. *Biol. Sex Differ.* 4:5. 10.1186/2042-6410-4-5 23514128 PMC3618244

[B49] Gabriele M. Lopez Tobon A. D’Agostino G. Testa G. (2018). The chromatin basis of neurodevelopmental disorders: rethinking dysfunction along the molecular and temporal axes. *Prog. Neuropsychopharmacol. Biol. Psychiatry* 84(Pt B), 306–327. 10.1016/j.pnpbp.2017.12.013 29309830

[B50] Gagnon J. Daou S. Zamorano N. Iannantuono N. V. Hammond-Martel I. Mashtalir N. (2015). Undetectable histone O-GlcNAcylation in mammalian cells. *Epigenetics* 10 677–691. 10.1080/15592294.2015.1060387 26075789 PMC4622518

[B51] Gambetta M. C. Müller J. (2014). O-GlcNAcylation prevents aggregation of the Polycomb group repressor polyhomeotic. *Dev. Cell* 31 629–639. 10.1016/j.devcel.2014.10.020 25468754

[B52] Gambetta M. C. Muller J. (2015). A critical perspective of the diverse roles of O-GlcNAc transferase in chromatin. *Chromosoma* 124 429–442. 10.1007/s00412-015-0513-1 25894967 PMC4666902

[B53] Gambetta M. C. Oktaba K. Muller J. (2009). Essential role of the glycosyltransferase sxc/Ogt in polycomb repression. *Science* 325 93–96. 10.1126/science.1169727 19478141

[B54] Gao J. Yang Y. Qiu R. Zhang K. Teng X. Liu R. (2018). Proteomic analysis of the OGT interactome: novel links to epithelial-mesenchymal transition and metastasis of cervical cancer. *Carcinogenesis* 39 1222–1234. 10.1093/carcin/bgy097 30052810 PMC6175026

[B55] Grassi D. A. Jönsson M. E. Brattås P. L. Jakobsson J. (2019). TRIM28 and the control of transposable elements in the brain. *Brain Res.* 1705 43–47. 10.1016/j.brainres.2018.02.043 29522722

[B56] Griffith L. S. Schmitz B. (1995). O-linked N-acetylglucosamine is upregulated in Alzheimer brains. *Biochem. Biophys. Res. Commun.* 213 424–431. 10.1006/bbrc.1995.2149 7646495

[B57] Guinez C. Filhoulaud G. Rayah-Benhamed F. Marmier S. Dubuquoy C. Dentin R. (2011). O-GlcNAcylation increases ChREBP protein content and transcriptional activity in the liver. *Diabetes* 60 1399–1413. 10.2337/db10-0452 21471514 PMC3292313

[B58] Hahne H. Gholami A. M. Kuster B. (2012). Discovery of O-GlcNAc-modified proteins in published large-scale proteome data. *Mol. Cell. Proteomics* 11 843–850. 10.1074/mcp.M112.019463 22661428 PMC3494142

[B59] Hajduskova M. Baytek G. Kolundzic E. Gosdschan A. Kazmierczak M. Ofenbauer A. (2019). MRG-1/MRG15 Is a Barrier for Germ Cell to Neuron Reprogramming in Caenorhabditis elegans. *Genetics* 211 121–139. 10.1534/genetics.118.301674 30425042 PMC6325694

[B60] Hanover J. Chen W. Bond M. (2018). O-GlcNAc in cancer: an Oncometabolism-fueled vicious cycle. *J. Bioenerg. Biomembr.* 50 155–173. 10.1007/s10863-018-9751-2 29594839

[B61] Hanover J. Krause M. Love D. (2012). Bittersweet memories: linking metabolism to epigenetics through O-GlcNAcylation. *Nat. Rev. Mol. Cell Biol.* 13 312–321. 10.1038/nrm3334 22522719

[B62] Hanover J. Yu S. Lubas W. Shin S. Ragano-Caracciola M. Kochran J. (2003). Mitochondrial and nucleocytoplasmic isoforms of O-linked GlcNAc transferase encoded by a single mammalian gene. *Arch. Biochem. Biophys.* 409 287–297. 10.1016/s0003-9861(02)00578-7 12504895

[B63] Hayakawa K. Hirosawa M. Tabei Y. Arai D. Tanaka S. Murakami N. (2013). Epigenetic switching by the metabolism-sensing factors in the generation of orexin neurons from mouse embryonic stem cells. *J. Biol. Chem.* 288 17099–17110. 10.1074/jbc.M113.455899 23625921 PMC3682516

[B64] Hayakawa K. Hirosawa M. Tani R. Yoneda C. Tanaka S. Shiota K. (2017). H2A O-GlcNAcylation at serine 40 functions genomic protection in association with acetylated H2AZ or gammaH2AX. *Epigenetics Chromatin* 10:51. 10.1186/s13072-017-0157-x 29084613 PMC5663087

[B65] He Y. F. Li B. Z. Li Z. Liu P. Wang Y. Tang Q. (2011). Tet-mediated formation of 5-carboxylcytosine and its excision by TDG in mammalian DNA. *Science* 333 1303–1307. 10.1126/science.1210944 21817016 PMC3462231

[B66] Herzig S. Long F. Jhala U. S. Hedrick S. Quinn R. Bauer A. (2001). CREB regulates hepatic gluconeogenesis through the coactivator PGC-1. *Nature* 413 179–183. 10.1038/35093131 11557984

[B67] Hirosawa M. Hayakawa K. Yoneda C. Arai D. Shiota H. Suzuki T. (2016). Novel O-GlcNAcylation on Ser(40) of canonical H2A isoforms specific to viviparity. *Sci. Rep.* 6:31785. 10.1038/srep31785 27615797 PMC5018834

[B68] Housley M. P. Rodgers J. T. Udeshi N. D. Kelly T. J. Shabanowitz J. Hunt D. F. (2008). O-GlcNAc regulates FoxO activation in response to glucose. *J. Biol. Chem.* 283 16283–16292. 10.1074/jbc.M802240200 18420577 PMC2423255

[B69] Howerton C. L. Bale T. L. (2012). Prenatal programing: at the intersection of maternal stress and immune activation. *Horm. Behav.* 62 237–242. 10.1016/j.yhbeh.2012.03.007 22465455 PMC3568743

[B70] Howerton C. L. Bale T. L. (2014). Targeted placental deletion of OGT recapitulates the prenatal stress phenotype including hypothalamic mitochondrial dysfunction. *Proc. Natl. Acad. Sci. U.S.A.* 111 9639–9644. 10.1073/pnas.1401203111 24979775 PMC4084439

[B71] Howerton C. L. Morgan C. P. Fischer D. B. Bale T. L. (2013). O-GlcNAc transferase (OGT) as a placental biomarker of maternal stress and reprogramming of CNS gene transcription in development. *Proc. Natl. Acad. Sci. U.S.A.* 110 5169–5174. 10.1073/pnas.1300065110 23487789 PMC3612602

[B72] Hrit J. Goodrich L. Li C. Wang B. A. Nie J. Cui X. (2018). OGT binds a conserved C-terminal domain of TET1 to regulate TET1 activity and function in development. *eLife* 7:e34870. 10.7554/eLife.34870 30325306 PMC6214653

[B73] Hu C. M. Tien S. C. Hsieh P. K. Jeng Y. M. Chang M. C. Chang Y. T. (2019). High Glucose Triggers Nucleotide Imbalance through O-GlcNAcylation of Key Enzymes and Induces KRAS Mutation in Pancreatic Cells. *Cell Metab.* 29 1334–1349.e10. 10.1016/j.cmet.2019.02.005 30853214

[B74] Imagawa E. Higashimoto K. Sakai Y. Numakura C. Okamoto N. Matsunaga S. (2017). Mutations in genes encoding polycomb repressive complex 2 subunits cause Weaver syndrome. *Hum. Mutat.* 38 637–648. 10.1002/humu.23200 28229514

[B75] Ingham P. W. (1984). A gene that regulates the bithorax complex differentially in larval and adult cells of Drosophila. *Cell* 37 815–823. 10.1016/0092-8674(84)90416-1 6430566

[B76] Ishihara K. Takahashi I. Tsuchiya Y. Hasegawa M. Kamemura K. (2010). Characteristic increase in nucleocytoplasmic protein glycosylation by O-GlcNAc in 3T3-L1 adipocyte differentiation. *Biochem. Biophys. Res. Commun.* 398 489–494. 10.1016/j.bbrc.2010.06.105 20599697

[B77] Ito R. Katsura S. Shimada H. Tsuchiya H. Hada M. Okumura T. (2014). TET3-OGT interaction increases the stability and the presence of OGT in chromatin. *Genes Cells* 19 52–65. 10.1111/gtc.12107 24304661

[B78] Ito S. D’Alessio A. C. Taranova O. V. Hong K. Sowers L. C. Zhang Y. (2010). Role of Tet proteins in 5mC to 5hmC conversion, ES-cell self-renewal and inner cell mass specification. *Nature* 466 1129–1133. 10.1038/nature09303 20639862 PMC3491567

[B79] Ito S. Shen L. Dai Q. Wu S. C. Collins L. B. Swenberg J. A. (2011). Tet proteins can convert 5-methylcytosine to 5-formylcytosine and 5-carboxylcytosine. *Science* 333 1300–1303. 10.1126/science.1210597 21778364 PMC3495246

[B80] Janetzko J. Trauger S. Lazarus M. Walker S. (2016). How the glycosyltransferase OGT catalyzes amide bond cleavage. *Nat. Chem. Biol.* 12 899–901. 10.1038/nchembio.2173 27618188 PMC5172607

[B81] Jang H. Kim T. W. Yoon S. Choi S. Y. Kang T. W. Kim S. Y. (2012). O-GlcNAc regulates pluripotency and reprogramming by directly acting on core components of the pluripotency network. *Cell Stem Cell* 11 62–74. 10.1016/j.stem.2012.03.001 22608532

[B82] Janke R. Dodson A. E. Rine J. (2015). Metabolism and epigenetics. *Annu. Rev. Cell Dev. Biol.* 31 473–496. 10.1146/annurev-cellbio-100814-125544 26359776 PMC5091661

[B83] Jenuwein T. Allis C. D. (2001). Translating the histone code. *Science* 293 1074–1080. 10.1126/science.1063127 11498575

[B84] Jiang M. Xu B. Li X. Shang Y. Chu Y. Wang W. (2019). O-GlcNAcylation promotes colorectal cancer metastasis via the miR-101-O-GlcNAc/EZH2 regulatory feedback circuit. *Oncogene* 38 301–316. 10.1038/s41388-018-0435-5 30093632 PMC6336687

[B85] Jinek M. Rehwinkel J. Lazarus B. D. Izaurralde E. Hanover J. A. Conti E. (2004). The superhelical TPR-repeat domain of O-linked GlcNAc transferase exhibits structural similarities to importin alpha. *Nat. Struct. Mol. Biol.* 11 1001–1007. 10.1038/nsmb833 15361863

[B86] Kapuria V. Röhrig U. Bhuiyan T. Borodkin V. van Aalten D. Zoete V. (2016). Proteolysis of HCF-1 by Ser/Thr glycosylation-incompetent O-GlcNAc transferase:UDP-GlcNAc complexes. *Genes Dev.* 30 960–972. 10.1101/gad.275925.115 27056667 PMC4840301

[B87] Kapuria V. Röhrig U. Waridel P. Lammers F. Borodkin V. van Aalten D. (2018). The conserved threonine-rich region of the HCF-1PRO repeat activates promiscuous OGT:UDP-GlcNAc glycosylation and proteolysis activities. *J. Biol. Chem.* 293 17754–17768. 10.1074/jbc.RA118.004185 30224358 PMC6240873

[B88] Katai E. Pal J. Poor V. S. Purewal R. Miseta A. Nagy T. (2016). Oxidative stress induces transient O-GlcNAc elevation and tau dephosphorylation in SH-SY5Y cells. *J. Cell. Mol. Med.* 20 2269–2277. 10.1111/jcmm.12910 27456536 PMC5134385

[B89] Keembiyehetty C. Love D. Harwood K. Gavrilova O. Comly M. Hanover J. (2015). Conditional knock-out reveals a requirement for O-linked N-Acetylglucosaminase (O-GlcNAcase) in metabolic homeostasis. *J. Biol. Chem.* 290 7097–7113. 10.1074/jbc.M114.617779 25596529 PMC4358131

[B90] Kim E. J. Amorelli B. Abdo M. Thomas C. J. Love D. C. Knapp S. (2007). Distinctive Inhibition of O-GlcNAcase Isoforms by an α-GlcNAc Thiolsulfonate. *J. Am. Chem. Soc.* 129 14854–14855. 10.1021/ja076038u 17994748

[B91] Kim G. Cao L. Reece E. A. Zhao Z. (2017). Impact of protein O-GlcNAcylation on neural tube malformation in diabetic embryopathy. *Sci. Rep.* 7:11107. 10.1038/s41598-017-11655-6 28894244 PMC5593976

[B92] Klose R. J. Bird A. P. (2006). Genomic DNA methylation: the mark and its mediators. *Trends Biochem. Sci.* 31 89–97. 10.1016/j.tibs.2005.12.008 16403636

[B93] Koufaris C. Alexandrou A. Tanteles G. A. Anastasiadou V. Sismani C. (2016). A novel HCFC1 variant in male siblings with intellectual disability and microcephaly in the absence of cobalamin disorder. *Biomed. Rep.* 4 215–218. 10.3892/br.2015.559 26893841 PMC4733959

[B94] Kriaucionis S. Heintz N. (2009). The nuclear DNA base 5-hydroxymethylcytosine is present in Purkinje neurons and the brain. *Science* 324 929–930. 10.1126/science.1169786 19372393 PMC3263819

[B95] Kuo M. Zilberfarb V. Gangneux N. Christeff N. Issad T. (2008). O-glycosylation of FoxO1 increases its transcriptional activity towards the glucose 6-phosphatase gene. *FEBS Lett.* 582 829–834. 10.1016/j.febslet.2008.02.010 18280254

[B96] Lamarre-Vincent N. Hsieh-Wilson L. C. (2003). Dynamic glycosylation of the transcription factor CREB: a potential role in gene regulation. *J. Am. Chem. Soc.* 125 6612–6613. 10.1021/ja028200t 12769553

[B97] Lambert B. Vandeputte J. Remacle S. Bergiers I. Simonis N. Twizere J. C. (2012). Protein interactions of the transcription factor Hoxa1. *BMC Dev. Biol.* 12:29. 10.1186/1471-213x-12-29 23088713 PMC3514159

[B98] Lane E. A. Choi D. W. Garcia-Haro L. Levine Z. G. Tedoldi M. Walker S. (2019). HCF-1 Regulates De Novo Lipogenesis through a Nutrient-Sensitive Complex with ChREBP. *Mol. Cell* 75 357–371.e7. 10.1016/j.molcel.2019.05.019 31227231 PMC6744259

[B99] Lazarus M. Jiang J. Kapuria V. Bhuiyan T. Janetzko J. Zandberg W. (2013). HCF-1 is cleaved in the active site of O-GlcNAc transferase. *Science* 342 1235–1239. 10.1126/science.1243990 24311690 PMC3930058

[B100] Lazarus M. B. Nam Y. Jiang J. Sliz P. Walker S. (2011). Structure of human O-GlcNAc transferase and its complex with a peptide substrate. *Nature* 469 564–567. 10.1038/nature09638 21240259 PMC3064491

[B101] Lercher L. Raj R. Patel N. A. Price J. Mohammed S. Robinson C. V. (2015). Generation of a synthetic GlcNAcylated nucleosome reveals regulation of stability by H2A-Thr101 GlcNAcylation. *Nat. Commun.* 6:7978. 10.1038/ncomms8978 26305776 PMC4560749

[B102] Leturcq M. Lefebvre T. Vercoutter-Edouart A. (2017). O-GlcNAcylation and chromatin remodeling in mammals: an up-to-date overview. *Biochem. Soc. Trans.* 45 323–338. 10.1042/BST20160388 28408473

[B103] Levine Z. Fan C. Melicher M. Orman M. Benjamin T. Walker S. (2018). O-GlcNAc Transferase Recognizes Protein Substrates Using an Asparagine Ladder in the Tetratricopeptide Repeat (TPR) Superhelix. *J. Am. Chem. Soc.* 140 3510–3513. 10.1021/jacs.7b13546 29485866 PMC5937710

[B104] Levine Z. Walker S. (2016). The Biochemistry of O-GlcNAc Transferase: Which Functions Make It Essential in Mammalian Cells. *Annu. Rev. Biochem.* 85 631–657. 10.1146/annurev-biochem-060713-035344 27294441

[B105] Lewis B. Hanover J. (2014). O-GlcNAc and the epigenetic regulation of gene expression. *J. Biol. Chem.* 289 34440–34448. 10.1074/jbc.R114.595439 25336654 PMC4263851

[B106] Li X. Molina H. Huang H. Zhang Y. Y. Liu M. Qian S. W. (2009). O-linked N-acetylglucosamine modification on CCAAT enhancer-binding protein beta: role during adipocyte differentiation. *J. Biol. Chem.* 284 19248–19254. 10.1074/jbc.M109.005678 19478079 PMC2740549

[B107] Li Z. Li X. Nai S. Geng Q. Liao J. Xu X. (2017). Checkpoint kinase 1-induced phosphorylation of O-linked beta-N-acetylglucosamine transferase regulates the intermediate filament network during cytokinesis. *J. Biol. Chem.* 292 19548–19555. 10.1074/jbc.M117.811646 29021254 PMC5712597

[B108] Liu C. Li J. (2018). O-GlcNAc: a sweetheart of the cell cycle and DNA Damage Response. *Front. Endocrinol.* 9:415. 10.3389/fendo.2018.00415 30105004 PMC6077185

[B109] Love D. Kochan J. Cathey R. Shin S. Hanover J. Kochran J. (2003). Mitochondrial and nucleocytoplasmic targeting of O-linked GlcNAc transferase. *J. Cell Sci.* 116(Pt 4), 647–654. 10.1242/jcs.00246 12538765

[B110] Love D. Krause M. Hanover J. (2010). O-GlcNAc cycling: emerging roles in development and epigenetics. *Semin. Cell Dev. Biol.* 21 646–654. 10.1016/j.semcdb.2010.05.001 20488252 PMC2917487

[B111] Lu S. Yin X. Wang J. Gu Q. Huang Q. Jin N. (2020). SIRT1 regulates O-GlcNAcylation of tau through OGT. *Aging* 12 7042–7055. 10.18632/aging.103062 32310828 PMC7202539

[B112] Ma D. K. Ming G. L. Song H. (2009). Oxysterols drive dopaminergic neurogenesis from stem cells. *Cell Stem Cell* 5 343–344. 10.1016/j.stem.2009.09.001 19796609 PMC6188706

[B113] Ma J. Hart G. W. (2014). O-GlcNAc profiling: from proteins to proteomes. *Clin. Proteomics* 11:8. 10.1186/1559-0275-11-8 24593906 PMC4015695

[B114] Maury J. J. El Farran C. A. Ng D. Loh Y. H. Bi X. Bardor M. (2015). RING1B O-GlcNAcylation regulates gene targeting of polycomb repressive complex 1 in human embryonic stem cells. *Stem Cell Res.* 15 182–189. 10.1016/j.scr.2015.06.007 26100231

[B115] Milne T. A. Sinclair D. A. Brock H. W. (1999). The Additional sex combs gene of Drosophila is required for activation and repression of homeotic loci, and interacts specifically with Polycomb and super sex combs. *Mol. Gen. Genet.* 261 753–761. 10.1007/s004380050018 10394912

[B116] Monfort A. Wutz A. (2020). The B-side of Xist. *F1000Res* 9:F1000 Faculty Rev-55 10.12688/f1000research.21362.1

[B117] Myers S. A. Panning B. Burlingame A. L. (2011). Polycomb repressive complex 2 is necessary for the normal site-specific O-GlcNAc distribution in mouse embryonic stem cells. *Proc. Natl. Acad. Sci. U.S.A.* 108 9490–9495. 10.1073/pnas.1019289108 21606357 PMC3111310

[B118] Na H.-J. Akan I. Abramowitz L. K. Hanover J. A. (2020). Nutrient-Driven O-GlcNAcylation Controls DNA Damage Repair Signaling and Stem/Progenitor Cell Homeostasis. *Cell Rep.* 31:107632. 10.1016/j.celrep.2020.107632 32402277 PMC9340802

[B119] Nishikawa I. Nakajima Y. Ito M. Fukuchi S. Homma K. Nishikawa K. (2010). Computational prediction of O-linked glycosylation sites that preferentially map on intrinsically disordered regions of extracellular proteins. *Int. J. Mol. Sci.* 11 4991–5008. 10.3390/ijms11124991 21614187 PMC3100847

[B120] Nugent B. M. Bale T. L. (2015). The omniscient placenta: metabolic and epigenetic regulation of fetal programming. *Front. Neuroendocrinol.* 39:28–37. 10.1016/j.yfrne.2015.09.001 26368654 PMC4681645

[B121] Nugent B. M. O’Donnell C. M. Epperson C. N. Bale T. L. (2018). Placental H3K27me3 establishes female resilience to prenatal insults. *Nat. Commun.* 9:2555. 10.1038/s41467-018-04992-1 29967448 PMC6028627

[B122] O’Donnell N. Zachara N. Hart G. Marth J. (2004). Ogt-dependent X-chromosome-linked protein glycosylation is a requisite modification in somatic cell function and embryo viability. *Mol. Cell. Biol.* 24 1680–1690. 10.1128/mcb.24.4.1680-1690.2004 14749383 PMC344186

[B123] Olivier-Van Stichelen S. Abramowitz L. Hanover J. (2014). X marks the spot: does it matter that O-GlcNAc transferase is an X-linked gene. *Biochem. Biophys. Res. Commun.* 453 201–207. 10.1016/j.bbrc.2014.06.068 24960196 PMC4253714

[B124] Olivier-Van Stichelen S. Hanover J. (2014). X-inactivation normalizes O-GlcNAc transferase levels and generates an O-GlcNAc-depleted Barr body. *Front. Genet.* 5:256. 10.3389/fgene.2014.00256 25136351 PMC4120696

[B125] Olivier-Van Stichelen S. Hanover J. (2015). You are what you eat: O-linked N-acetylglucosamine in disease, development and epigenetics. *Curr. Opin. Clin. Nutr. Metab. Care* 18 339–345. 10.1097/MCO.0000000000000188 26049631 PMC4479189

[B126] Olivier-Van Stichelen S. Wang P. Comly M. Love D. Hanover J. (2017). Nutrient-driven O-linked N-acetylglucosamine (O-GlcNAc) cycling impacts neurodevelopmental timing and metabolism. *J. Biol. Chem.* 292 6076–6085. 10.1074/jbc.M116.774042 28246173 PMC5391740

[B127] Park S. Zhou X. Pendleton K. Hunter O. Kohler J. O’Donnell K. (2017). A Conserved Splicing Silencer Dynamically Regulates O-GlcNAc Transferase Intron Retention and O-GlcNAc Homeostasis. *Cell Rep.* 20 1088–1099. 10.1016/j.celrep.2017.07.017 28768194 PMC5588854

[B128] Parra M. Zhang W. Vu J. DeWitt M. Conboy J. (2020). Antisense targeting of decoy exons can reduce intron retention and increase protein expression in human erythroblasts. *RNA* 26 996–1005. 10.1261/rna.075028.120 32312846 PMC7373989

[B129] Pick H. Kilic S. Fierz B. (2014). Engineering chromatin states: chemical and synthetic biology approaches to investigate histone modification function. *Biochim. Biophys. Acta* 1839 644–656. 10.1016/j.bbagrm.2014.04.016 24768924

[B130] Pravata V. Gundogdu M. Bartual S. Ferenbach A. Stavridis M. Õunap K. (2020a). A missense mutation in the catalytic domain of O-GlcNAc transferase links perturbations in protein O-GlcNAcylation to X-linked intellectual disability. *FEBS Lett.* 594 717–727. 10.1002/1873-3468.13640 31627256 PMC7042088

[B131] Pravata V. Muha V. Gundogdu M. Ferenbach A. Kakade P. Vandadi V. (2019). Catalytic deficiency of O-GlcNAc transferase leads to X-linked intellectual disability. *Proc. Natl. Acad. Sci. U.S.A.* 116 14961–14970. 10.1073/pnas.1900065116 31296563 PMC6660750

[B132] Pravata V. Omelková M. Stavridis M. Desbiens C. Stephen H. Lefeber D. (2020b). An intellectual disability syndrome with single-nucleotide variants in O-GlcNAc transferase. *Eur. J. Hum. Genet.* 28 706–714. 10.1038/s41431-020-0589-9 32080367 PMC7253464

[B133] Quinonez S. C. Innis J. W. (2014). Human HOX gene disorders. *Mol. Genet. Metab.* 111 4–15. 10.1016/j.ymgme.2013.10.012 24239177

[B134] Rahe D. P. Hobert O. (2019). Restriction of Cellular Plasticity of Differentiated Cells Mediated by Chromatin Modifiers, Transcription Factors and Protein Kinases. *G3* 9 2287–2302. 10.1534/g3.119.400328 31088904 PMC6643894

[B135] Rao F. V. Schuttelkopf A. W. Dorfmueller H. C. Ferenbach A. T. Navratilova I. van Aalten D. M. (2013). Structure of a bacterial putative acetyltransferase defines the fold of the human O-GlcNAcase C-terminal domain. *Open Biol.* 3:130021. 10.1098/rsob.130021 24088714 PMC3814719

[B136] Rexach J. E. Clark P. M. Mason D. E. Neve R. L. Peters E. C. Hsieh-Wilson L. C. (2012). Dynamic O-GlcNAc modification regulates CREB-mediated gene expression and memory formation. *Nat. Chem. Biol.* 8 253–261. 10.1038/nchembio.770 22267118 PMC3288555

[B137] Ronningen T. Shah A. Oldenburg A. R. Vekterud K. Delbarre E. Moskaug J. O. (2015). Prepatterning of differentiation-driven nuclear lamin A/C-associated chromatin domains by GlcNAcylated histone H2B. *Genome Res.* 25 1825–1835. 10.1101/gr.193748.115 26359231 PMC4665004

[B138] Rowe L. A. Degtyareva N. Doetsch P. W. (2008). DNA damage-induced reactive oxygen species (ROS) stress response in Saccharomyces cerevisiae. *Free Radic. Biol. Med.* 45 1167–1177. 10.1016/j.freeradbiomed.2008.07.018 18708137 PMC2643028

[B139] Saeed M. Ahmad J. Kanwal S. Holowatyj A. Sheikh I. Zafar Paracha R. (2016). Formal modeling and analysis of the hexosamine biosynthetic pathway: role of O-linked N-acetylglucosamine transferase in oncogenesis and cancer progression. *PeerJ* 4:e2348. 10.7717/peerj.2348 27703839 PMC5047222

[B140] Sakabe K. Wang Z. Hart G. W. (2010). Beta-N-acetylglucosamine (O-GlcNAc) is part of the histone code. *Proc. Natl. Acad. Sci. U.S.A.* 107 19915–19920. 10.1073/pnas.1009023107 21045127 PMC2993388

[B141] SanMiguel J. M. Bartolomei M. S. (2018). DNA methylation dynamics of genomic imprinting in mouse development. *Biol. Reprod.* 99 252–262. 10.1093/biolre/ioy036 29462489 PMC6044325

[B142] Savage J. E. Jansen P. R. Stringer S. Watanabe K. Bryois J. de Leeuw C. A. (2018). Genome-wide association meta-analysis in 269,867 individuals identifies new genetic and functional links to intelligence. *Nat. Genet.* 50 912–919. 10.1038/s41588-018-0152-6 29942086 PMC6411041

[B143] Schouppe D. Ghesquiere B. Menschaert G. De Vos W. H. Bourque S. Trooskens G. (2011). Interaction of the tobacco lectin with histone proteins. *Plant Physiol.* 155 1091–1102. 10.1104/pp.110.170134 21224338 PMC3046571

[B144] Schvartzman J. M. Thompson C. B. Finley L. W. S. (2018). Metabolic regulation of chromatin modifications and gene expression. *J. Cell Biol.* 217 2247–2259. 10.1083/jcb.201803061 29760106 PMC6028552

[B145] Selvan N. George S. Serajee F. Shaw M. Hobson L. Kalscheuer V. (2018). *O*-GlcNAc transferase missense mutations linked to X-linked intellectual disability deregulate genes involved in cell fate determination and signaling. *J. Biol. Chem.* 293 10810–10824. 10.1074/jbc.RA118.002583 29769320 PMC6036218

[B146] Shafi R. Iyer S. Ellies L. O’Donnell N. Marek K. Chui D. (2000). The O-GlcNAc transferase gene resides on the X chromosome and is essential for embryonic stem cell viability and mouse ontogeny. *Proc. Natl. Acad. Sci. U.S.A.* 97 5735–5739. 10.1073/pnas.100471497 10801981 PMC18502

[B147] Shi F. T. Kim H. Lu W. He Q. Liu D. Goodell M. A. (2013a). Ten-eleven translocation 1 (Tet1) is regulated by O-linked N-acetylglucosamine transferase (Ogt) for target gene repression in mouse embryonic stem cells. *J. Biol. Chem.* 288 20776–20784. 10.1074/jbc.M113.460386 23729667 PMC3774349

[B148] Shi X. Sun M. Liu H. Yao Y. Song Y. (2013b). Long non-coding RNAs: a new frontier in the study of human diseases. *Cancer Lett.* 339 159–166. 10.1016/j.canlet.2013.06.013 23791884

[B149] Sinclair D. A. Syrzycka M. Macauley M. S. Rastgardani T. Komljenovic I. Vocadlo D. J. (2009). Drosophila O-GlcNAc transferase (OGT) is encoded by the Polycomb group (PcG) gene, super sex combs (sxc). *Proc. Natl. Acad. Sci. U.S.A.* 106 13427–13432. 10.1073/pnas.0904638106 19666537 PMC2726349

[B150] Tahiliani M. Koh K. P. Shen Y. Pastor W. A. Bandukwala H. Brudno Y. (2009). Conversion of 5-methylcytosine to 5-hydroxymethylcytosine in mammalian DNA by MLL partner TET1. *Science* 324 930–935. 10.1126/science.1170116 19372391 PMC2715015

[B151] Tan Z. Fei G. Paulo J. Bellaousov S. Martin S. Duveau D. (2020). O-GlcNAc regulates gene expression by controlling detained intron splicing. *Nucleic Acids Res.* 48 5656–5669. 10.1093/nar/gkaa263 32329777 PMC7261177

[B152] Toleman C. A. Paterson A. J. Kudlow J. E. (2006). The Histone Acetyltransferase NCOAT Contains a Zinc Finger-like Motif Involved in Substrate Recognition. *J. Biol. Chem.* 281 3918–3925. 10.1074/jbc.m510485200 16356930

[B153] Torres C. R. Hart G. W. (1984). Topography and polypeptide distribution of terminal N-acetylglucosamine residues on the surfaces of intact lymphocytes. Evidence for O-linked GlcNAc. *J. Biol. Chem.* 259 3308–3317. 6421821

[B154] Tursun B. Patel T. Kratsios P. Hobert O. (2011). Direct conversion of C. elegans germ cells into specific neuron types. *Science* 331 304–308. 10.1126/science.1199082 21148348 PMC3250927

[B155] Vaidyanathan K. Niranjan T. Selvan N. Teo C. May M. Patel S. (2017). Identification and characterization of a missense mutation in the O-linked β-N-acetylglucosamine (O-GlcNAc) transferase gene that segregates with X-linked intellectual disability. *J. Biol. Chem.* 292 8948–8963. 10.1074/jbc.M116.771030 28302723 PMC5448127

[B156] Vella P. Scelfo A. Jammula S. Chiacchiera F. Williams K. Cuomo A. (2013). Tet proteins connect the O-linked N-acetylglucosamine transferase Ogt to chromatin in embryonic stem cells. *Mol. Cell* 49 645–656. 10.1016/j.molcel.2012.12.019 23352454

[B157] Wahl G. M. Carr A. M. (2001). The evolution of diverse biological responses to DNA damage: insights from yeast and p53. *Nat. Cell Biol.* 3 E277–E286. 10.1038/ncb1201-e277 11781586

[B158] Whitelaw N. C. Chong S. Morgan D. K. Nestor C. Bruxner T. J. Ashe A. (2010). Reduced levels of two modifiers of epigenetic gene silencing, Dnmt3a and Trim28, cause increased phenotypic noise. *Genome Biol.* 11:R111. 10.1186/gb-2010-11-11-r111 21092094 PMC3156950

[B159] Willems A. Gundogdu M. Kempers M. Giltay J. Pfundt R. Elferink M. (2017). Mutations in N-acetylglucosamine (O-GlcNAc) transferase in patients with X-linked intellectual disability. *J. Biol. Chem.* 292 12621–12631. 10.1074/jbc.M117.790097 28584052 PMC5535036

[B160] Wong C. C. Qian Y. Yu J. (2017). Interplay between epigenetics and metabolism in oncogenesis: mechanisms and therapeutic approaches. *Oncogene* 36 3359–3374. 10.1038/onc.2016.485 28092669 PMC5485177

[B161] Xie S. Jin N. Gu J. Shi J. Sun J. Chu D. (2016). O-GlcNAcylation of protein kinase A catalytic subunits enhances its activity: a mechanism linked to learning and memory deficits in Alzheimer’s disease. *Aging Cell* 15 455–464. 10.1111/acel.12449 26840030 PMC4854926

[B162] Yang X. Qian K. (2017). Protein O-GlcNAcylation: emerging mechanisms and functions. *Nat. Rev. Mol. Cell Biol.* 18 452–465. 10.1038/nrm.2017.22 28488703 PMC5667541

[B163] Yang X. Zhang F. Kudlow J. E. (2002). Recruitment of O-GlcNAc transferase to promoters by corepressor mSin3A: coupling protein O-GlcNAcylation to transcriptional repression. *Cell* 110 69–80. 10.1016/s0092-8674(02)00810-3 12150998

[B164] Zhang Q. Liu X. Gao W. Li P. Hou J. Li J. (2014). Differential regulation of the ten-eleven translocation (TET) family of dioxygenases by O-linked beta-N-acetylglucosamine transferase (OGT). *J. Biol. Chem.* 289 5986–5996. 10.1074/jbc.M113.524140 24394411 PMC3937666

[B165] Zhang S. Roche K. Nasheuer H. P. Lowndes N. F. (2011). Modification of histones by sugar beta-N-acetylglucosamine (GlcNAc) occurs on multiple residues, including histone H3 serine 10, and is cell cycle-regulated. *J. Biol. Chem.* 286 37483–37495. 10.1074/jbc.M111.284885 21896475 PMC3199494

